# A phosphatase-like nanomaterial promotes autophagy and reprograms macrophages for cancer immunotherapy†

**DOI:** 10.1039/d4sc01690d

**Published:** 2024-06-17

**Authors:** Didar Baimanov, Su Li, Xuejiao J. Gao, Rui Cai, Ke Liu, Junjie Li, Yuchen Liu, Yalin Cong, Xiaoyu Wang, Fen Liu, Qi Li, Guofang Zhang, Hui Wei, Jian Wang, Chunying Chen, Xingfa Gao, Yang Li, Liming Wang

**Affiliations:** a CAS Key Laboratory for Biomedical Effects of Nanomaterials and Nanosafety, CAS-HKU Joint Laboratory of Metallomics on Health and Environment, Institute of High Energy Physics, Chinese Academy of Sciences and National Center for Nanoscience and Technology of China Beijing 100049 P. R. China wangliming@ihep.ac.cn; b Laboratory of Immunology and Nanomedicine & China-Italy Joint Laboratory of Pharmacobiotechnology for Medical Immunomodulation, Laboratory of Inflammation and Vaccines, Shenzhen Institute of Advanced Technology, Chinese Academy of Sciences Shenzhen 518055 P. R. China yang.li@siat.ac.cn; c Division of Allergy & Immunology, Department of Biosciences & Medical Biology, Paris Lodron University of Salzburg 5020 Salzburg Austria; d College of Chemistry and Chemical Engineering, Jiangxi Normal University Nanchang 330022 P. R. China; e State Key Laboratory of Medical Proteomics, Beijing Proteome Research Center, National Center for Protein Sciences (Beijing), Beijing Institute of Lifeomics Beijing 102206 P. R. China; f College of Engineering and Applied Sciences, Nanjing National Laboratory of Microstructures, Jiangsu Key Laboratory of Artificial Functional Materials, Nanjing University Nanjing 210093 P. R. China; g State Key Laboratory of Natural and Biomimetic Drugs, School of Pharmaceutical Sciences, Peking University Beijing 100191 P. R. China; h Laboratory of Theoretical and Computational Nanoscience, National Center for Nanoscience and Technology of China Beijing 100190 P. R. China gaoxf@nanoctr.cn; i New Cornerstone Science Laboratory, National Center for Nanoscience and Technology of China Beijing 100049 P. R. China; j The Key Laboratory of Biomedical Imaging Science and System, Chinese Academy of Sciences Shenzhen P. R. China

## Abstract

Macrophages are plastic and play a key role in the maintenance of tissue homeostasis. In cancer progression, macrophages also take part in all processes, from initiation to progression, to final tumor metastasis. Although energy deprivation and autophagy are widely used for cancer therapy, most of these strategies do not target macrophages, resulting in undesired effects and unsatisfactory outcomes for cancer immunotherapy. Herein, we developed a lanthanum nickel oxide (LNO) nanozyme with phosphatase-like activity for ATP hydrolysis. Meanwhile, the autophagy of macrophages induced by LNO promotes the polarization of macrophages from M2-like macrophages (M2) to M1-like macrophages (M1) and reduces tumor-associated macrophages in tumor-bearing mice, exhibiting the capability of killing tumor-associated macrophages and antitumor effects *in vivo*. Furthermore, pre-coating the surface of LNO with a myeloid cell membrane significantly enhanced antitumor immunity. Our findings demonstrate that phosphatase-like nanozyme LNO can specifically induce macrophage autophagy, which improves therapeutic efficacy and offers valuable strategies for cancer immunotherapy.

## Abstract 2

Phosphatase-like nanozyme LNO specifically induces macrophage autophagy, improving antitumor immunity. Pre-coating LNO with myeloid cell membranes further enhances this effect, demonstrating significant therapeutic efficacy *in vivo*.

## Graphical abstract

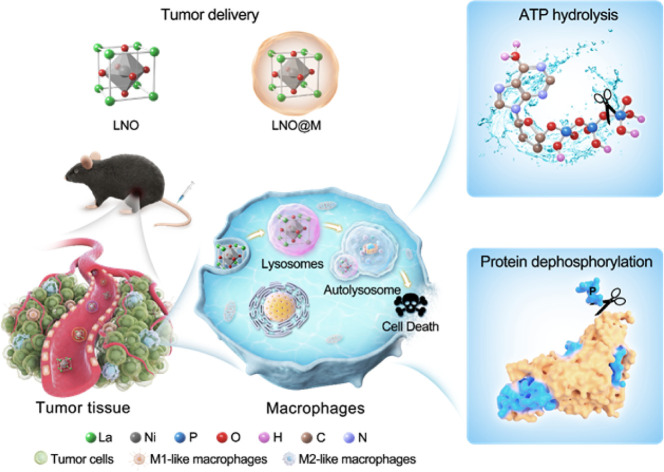

## Introduction

Macrophages are immune surveillance cells and phagocytes belonging to the innate immune system. Macrophages are responsible for managing dangerous substances, including pathogens and dangerous endogenous factors, to maintain homeostasis in the body and serve as an important part of the body's first line of immune responses. Macrophages also play a key role in cancer progression through all the different tumor stages, from initiation to progression, to final tumor metastasis. Macrophages are double-edged swords in the tumor microenvironment (TME). By secreting various immune factors, macrophages can crosstalk with other cells in the TME, thereby supporting or suppressing cancer development.^1,2^ Tumor-associated macrophages (TAMs) are generally M2-like phenotypes that suppress antitumor immune responses for tumor growth.^3,4^ In contrast, M1-like macrophages can destroy malignant tumor cells *via* phagocytosis, which is normally suppressed in the TME. Considering their important roles in antitumor therapeutic strategies, macrophages have become a promising target for cancer immunotherapy.^5^ Therefore, the development of targeted therapies that effectively reprogram macrophages in the TME is a promising direction and is currently being developed with great efforts.

Autophagy is a critical regulatory process in macrophages that not only maintains cellular homeostasis but also plays a role in specific immune functions^6,7^ and the development of cardiovascular diseases, neurodegenerative diseases, the immune system, and cancer,^8,9^ which are also involved in regulating and maintaining normal physiological functions.^10^ Adenosine triphosphate (ATP)^11^ depletion mediates energy deprivation conditions that activates AMP-activated protein kinase (AMPK)^12^ and is involved in autophagy induction.^13^ AMPK pathways leading to reduced cell proliferation act through inhibition of mammalian target of rapamycin complex 1 (mTORC1) activity.^14^ mTOR is inactivated during nutrient shortages, allowing autophagosome formation and activation of lysosomes through autophagic lysosome reformation process.^15^ When nutrients are abundant, mTORC1 promotes protein synthesis *via* the phosphorylation of substrates implicated in translation regulation and inhibits autophagy at multiple levels by inactivating proteins involved in autophagosome formation.^16^ Therefore, the development of nanomedicines that can trigger macrophage autophagy is an attractive strategy for cancer immunotherapy. With regard to multifunctionality, nanomedicines combining intracellular protein dephosphorylation with ATP hydrolysis, which specifically trigger macrophage autophagy, will be promising for cancer immunotherapy.

Previous studies have indicated that the efficacy of lanthanide nanoparticles for oncotherapy was conducted at the cellular level at high dosages, which could induce cancer cell death^17^*via* autophagy activation^18^ and IL-1β production,^19,20^ causing cell membrane permeabilization.^21^ However, little is known about the therapeutic effects of lanthanide nanoparticles from the chemical and physical aspects at the molecular level and at the interface between nanoparticles and biological molecules. Considering that nanoparticles might exhibit toxicity at high doses and off-target delivery, nanoparticles with higher target delivery at lower dose efficacy should be developed to reduce the existing side effects of therapeutics. In addition, *in vitro* results in cancer cells are often difficult to translate *in vivo* because of the lack of delivery efficacy and sufficient clearance by myeloid cells. In contrast, we focused on the design of lanthanum-based nanoparticles or nanomedicines with low toxicity to normal cells, specific interaction with macrophages, and increased accumulation in tumor tissues for potential immunotherapeutic applications. For cancer immunotherapy, it is crucial to fabricate nanomedicines that are capable of targeting tumor-associated macrophages, regulating their interactions with intracellular biomolecules, and resulting in macrophage autophagy.

**Scheme 1:**
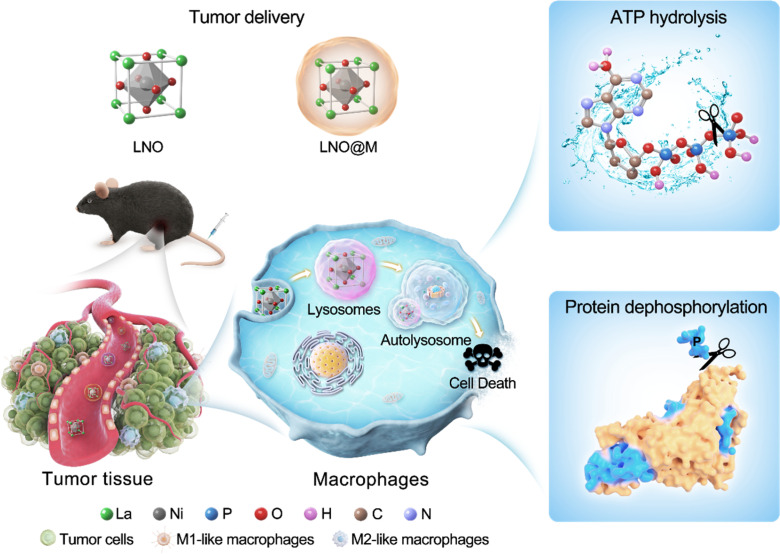
Schematic illustration of the mechanism by which LNO nanoparticles boost antitumor immunotherapy by ATP hydrolysis and protein dephosphorylation, leading to the autophagy in macrophages. Myeloid cell membrane-coated LNO nanoparticles are injected into tumor-bearing mice that are marked as LNO@M.

Herein, we propose a strategy that combines energy modulation with selectively induced autophagy of macrophages using lanthanide-based metal oxide nanoparticles (LaNiO_3_, LNO) that efficiently reprograms macrophages during antitumor responses. Nickel and oxygen atoms on the surface of LNO play a crucial role in the transformation and catalytic activities of ATP hydrolysis and intracellular protein dephosphorylation by sequestration of phosphates, leading to energy deprivation and autophagy in macrophages. Moreover, cell membrane-coated LNO-mediated cancer immunotherapy has been effective in targeting the TME and is recognized by macrophages, reducing the amount of M2 macrophages and resulting in macrophage reprogramming and tumor suppression *in vivo*, suggesting its potential use in cancer treatment. Based on LNO-mediated macrophage autophagy, we used cell membrane-coated LNO to evaluate the efficiency of cancer immunotherapy (Scheme 1). Overall, our study introduces a novel approach to nanomedicine with phosphatase-like activity, including ATP hydrolysis and protein dephosphorylation capabilities, which eventually promotes macrophage autophagy and holds promise for future cancer immunotherapy. These findings have significant implications for the development of cancer immunotherapy.

## Results and discussion

### Molecular and intracellular ATP hydrolysis by LNO

**Fig. 1:**
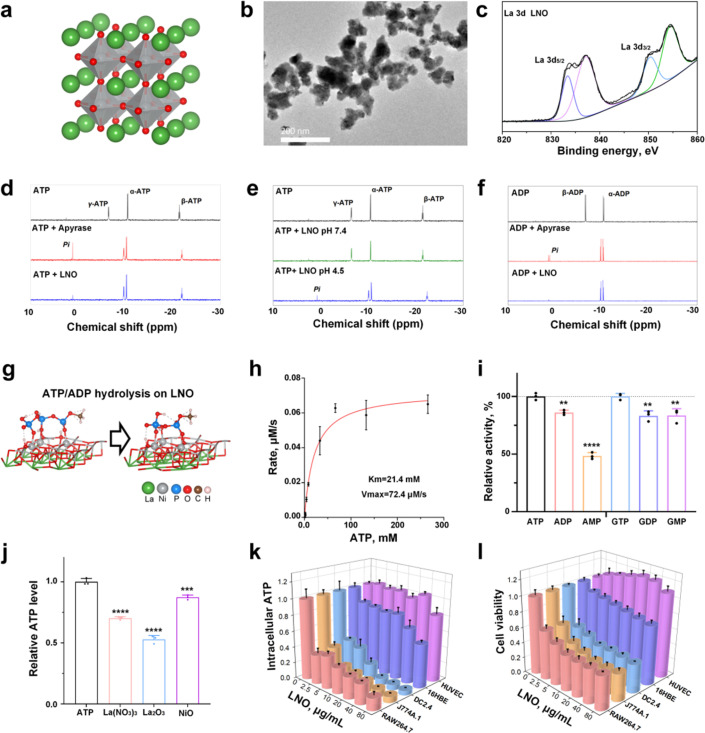
Hydrolysis mechanism of phosphate-containing biological molecules by LNO. (a) Schematic illustration and (b) TEM image of LNO. The scale bar represents 200 nm. (c) XPS spectrum of La 3d in LNO. The binding energies of La 3d_5/2_ and La 3d_3/2_ peaks are present at 833.2 and 850.2 eV. (d–f) ^31^P-NMR spectra of the catalytic hydrolysis of ATP (d, e) and ADP (f) by LNO and apyrase. ^31^P-NMR spectra of ATP and ADP solutions and their hydrolyzed samples are obtained by incubating ATP or ADP solution with apyrase at pH 7.4, and LNO at pH 4.5 and 7.4, respectively. (g) Schematic illustration of ATP/ADP hydrolysis by LNO. (h) Michaelis–Menten plots for LNO at various ATP concentrations. (i) Relative activity of LNO toward the catalytic hydrolysis of various phosphate-bond-containing substrates. The hydrolytic activity of LNO toward ATP and GTP is presented as 100%. (j) Catalytic activities of La(NO_3_)_3_, La_2_O_3_, and NiO aqueous solutions toward ATP hydrolysis. (k and l) Intracellular ATP levels (k) and cytotoxicity (l) of LNO in different cell lines, including macrophages, dendritic cells, and other cells after 24 h treatment with different doses of LNO. Statistical significance is calculated using one-way ANOVA with Tukey's multiple comparison test; **p < 0.01; ***p < 0.001; ****p < 0.0001. Data are expressed as the mean ± standard deviation for replicated samples (*n* = 3 for (h–k), and *n* = 5 for (l)).

The structure of the engineered LNO is shown in Fig. 1a. LNO^22^ (Fig. 1b) was characterized by various techniques such as transmission electron microscopy (TEM), zeta potential analysis, and X-ray photoelectron spectroscopy (XPS) that analyzed its size, morphology, surface, and structural properties (Fig. 1c and ESI Fig. S1†). Elemental identification and valence state analysis of LNO were performed by XPS (Fig. 1c and S1a, b†). The surface charges of LNO were measured at pH 7.4 and pH 4.5 which exhibited −12.5 mV and −3.8 mV, respectively (Fig. S1c†).

Next, we measured the catalytic activity of LNO on biomolecules, such as ATP and GTP. We first attempted to identify the factors that influence ATP hydrolysis and catalytic activity of LNO (Fig. S2†). The results showed efficient hydrolysis of ATP by LNO under acidic conditions (Fig. S2a and b†) in a concentration-dependent manner (Fig. S2c†). We concluded that LNO acts as an acidic phosphatase-like nanozyme and is most effective at an optimal pH of 4.5 at 37 °C. To further investigate the mechanism of LNO's catalytic activity, we compared the hydrolysis of ATP and ADP by LNO and apyrase under acidic and physiological conditions (Fig. 1d–f and S3†). The results showed that both LNO and apyrase decreased the characteristic peaks of γ-ATP and β-ATP within 30 min of incubation. We observed that the characteristic peaks of γ-ATP (−6.4 ppm) and β-ADP (−6.4 ppm) disappeared within 30 min of incubation with LNO and apyrase. Interestingly, we observed a phosphate peak (1.6 ppm) during hydrolysis by apyrase. These findings suggest that LNO functions similarly to the apyrase enzyme by cleaving γ-ATP or β-ADP but without producing detectable amounts of free phosphate (Pi). To ensure efficient ATP hydrolysis over LNO (Fig. 1g), the catalytic activity of LNO was calculated using the Michaelis–Menten curve (Fig. 1h). Importantly, we also observed that LNO could hydrolyze various phosphate bond-containing substrates such as ADP, AMP, and GTP, *etc.* (Fig. 1i). To compare the hydrolytic activity of LNO, several metal ions were tested (Fig. 1j), and the results showed that LNO was a more effective phosphatase-like nanozyme for biotechnological applications.

We hypothesized that LNO might have a strong impact on the phosphate groups (*i.e.*, intracellular ATP) acting as a nanozyme,^23^ resulting in ATP hydrolysis. We assumed that immune cells might undergo energy deprivation upon interaction with LNO, significantly affecting macrophage viability. Likewise, we observed a dramatic decrease in intracellular ATP of macrophages, dendritic (DC) cells and neglectable alterations in other types of cells (Fig. 1k and S4, S5†). We observed that LNO significantly affects the viability of macrophages at low concentrations (Fig. 1l and S4†).

It has been suggested that LNO can induce cytotoxicity in macrophages and DC, providing an opportunity for cancer immunotherapy by targeting macrophages. To further investigate this concern, we applied the LDH assay, as the release of LDH molecules is triggered upon disruption of the cell membrane due to cell death. Similarly, we found that LNO induced a dramatic release of LDH from the immune cells (Fig. S4 and S6†). Taken together, our results revealed that LNO may significantly hydrolyze intracellular ATP in a concentration-dependent manner, leading to energy deprivation in macrophages.

### Nickel and oxygen active sites of LNO for ATP hydrolysis

**Fig. 2:**
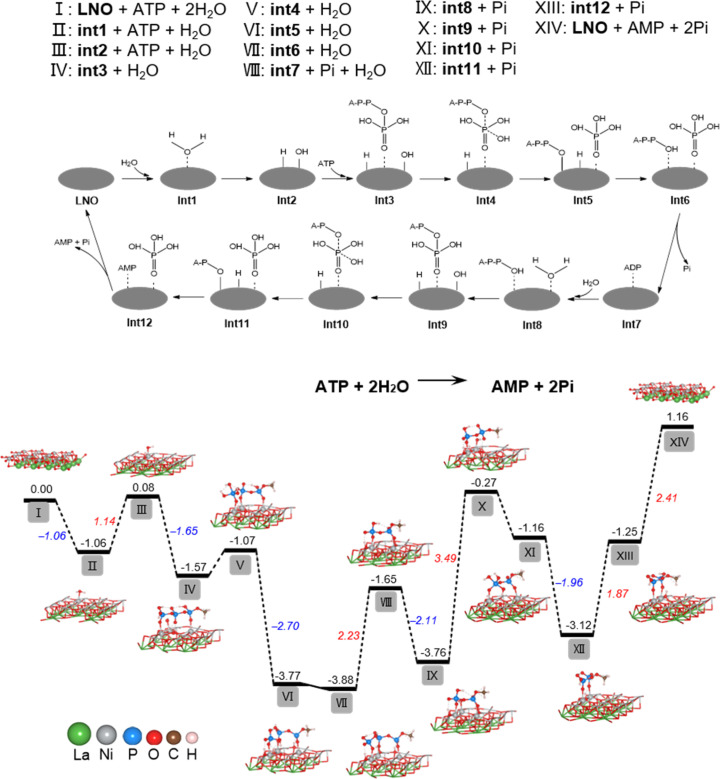
The free energy profile for the process of ATP hydrolysis by LNO based on DFT simulation. The proposed mechanism for the hydrolysis of ATP on the surface of LNO is represented by an equation and grey ellipse. The intermediates involved are marked as int1–int12. The calculated free energy profiles (eV) correspond to the mechanism and chemical constituents of stationary points I–XIV. Intermediate geometric structures are also illustrated.

To determine the underlying mechanism of ATP hydrolysis, density functional theory (DFT) calculations were conducted (Fig. 2). LNO was chosen as the model material for this study due to the best POD-mimicking performance,^22^ attributed to its ability to break the O–O bond and form the key intermediate, adsorbed HO*, which is also pivotal in our current work. The NiO_2_ termination of the LaNiO_3_ (001) surface was selected as the surfaces of reactions, because transition metal Ni atoms in these surfaces are all five coordinated and each has one open coordination site. The five coordinated NiO_2_ termination is analogous to metals in metalloporphyrins, the active centers of many natural enzymes. Besides, our previous work demonstrated that the intrinsic peroxidase-like activity of ABO_3_ is sensitive to the change of B atom instead of A.^22^ Also, other researchers used the BO_2_ termination as the active surface for other reactions like the OER and ORR.^24–26^ We designed a two-step hydrolysis process from ATP to ADP and then to AMP on the surface of LNO, which consumes two equivalent water molecules and releases two equivalent phosphoric acids (Pi), involving 12 intermediates (int1–int12) in the cycle. First, the adsorption of H_2_O to LNO surface with a free energy release (−1.06 eV), with further breakage of the O–H bond (II–III), was accompanied by an energy increase to 1.14 eV and generation of H* and OH* (* indicates adsorbed state). Furthermore, the adsorption of ATP onto LNO (*G*_ads_, ATP = −1.65 eV) (III–IV) indicates a strong interaction between ATP and LNO. The OH* connects to the P atom of the outermost Pi, generating int4 with a relatively small energy increase (IV–V). Subsequently, the P–O bond breaks with a notable energy release, generating int5 with Pi* (V–VI). The H* automatically bonds with the dangling O terminal to form int6, which consists of ADP* and Pi*, while the desorption of Pi requires a relatively high energy release (2.23 eV). Then, the second water molecule joins the reaction with ADP* (int7), hydrolyzes the outermost P–O bond of ADP through a similar path (VIII–XIII), and finally generates AMP* and Pi* (int12). In contrast to the first step of hydrolysis, the breaking of the O–H bond of water (IX–X) and the binding of H to A–P–O (XII–XIII) require overcoming higher energies (3.49 and 1.87 eV, respectively). The above results show that LNO can effectively catalyze the hydrolysis of ATP phosphate bonds, whereas the hydrolysis of ADP phosphate bonds is much more difficult than that of ATP. The DFT calculations agreed well with the experimental observations and explained why less free Pi was detected because of the strong adsorption of Pi on LNO (*G*_ads_, Pi = −2.41 eV).

It has been shown that the metal–oxygen bonds in perovskites have mixed ionic-covalent character due to the similar covalency and spatial overlap of metal and oxygen orbitals influencing catalytic activities of perovskites. Therefore, as highly covalent late-transition-metal perovskites, LNO can have both metal and oxygen as active sites, and in some cases, can exhibit pH-dependent oxygen evolution reaction activity involving proton-electron transfers in catalytic processes.^27,28^ Therefore, our findings are supported by DFT calculations for ATP hydrolysis mechanisms, showing that both nickel and oxygen act as active sites for LNO perovskites, resulting in phosphate sequestration.

### Intracellular protein dephosphorylations lead to sequential autophagy activation

**Fig. 3:**
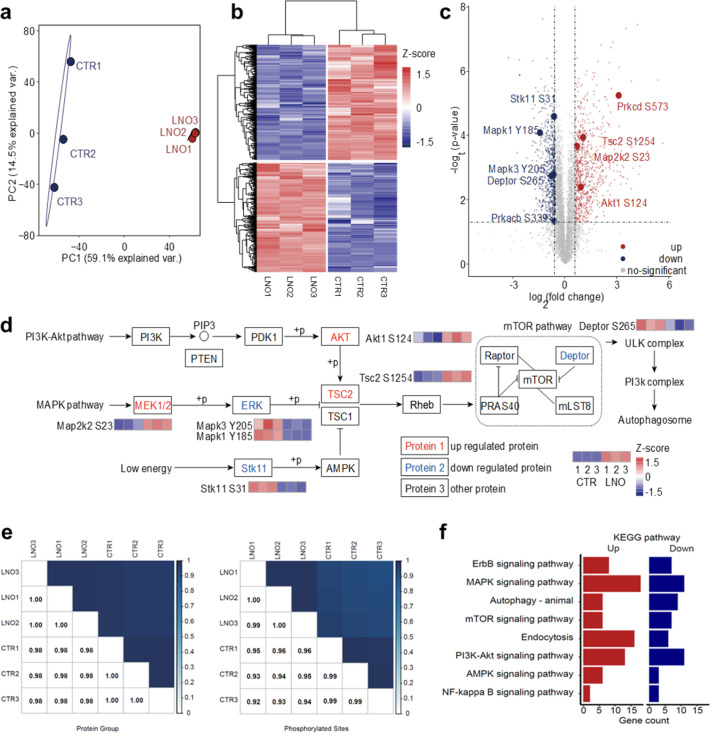
Impact of LNO on the cellular phosphoproteins. (a) Principal component analysis (PCA) map of phosphorylation proteomes from LNO-treated and untreated RAW264.7 cell line. The level of phosphorylated proteins changes after LNO treatment compared to that in untreated cells. (b) Hierarchical clustering of *Z*-scores derived from differentially expressed proteins between LNO treatment and control in triplicate cultures. (c) Volcano plot showing the phosphoproteome with and without LNO treatment. Significantly regulated phosphosites (*t*-test) are indicated in red (upregulated) or blue (downregulated), *p* < 0.05, and absolute fold-change >1.5. Phosphorylated proteins involved in autophagy-related pathways are labelled with darker colour. The unadjusted *p*-value was used for differential phosphorylated proteome analysis. (d) Analysis of phosphosites on proteins in the autophagy signalling network and several upstream pathways, including PI3K-Akt, MAPK, AMPK, and mTOR. Phosphosites with significant changes are labelled using *Z*-score of biological triplicates. (e) Pearson correlation coefficients of protein group and phosphorylation abundance data. (f) List of up- and down-regulated KEGG pathways in LNO-treated group compared to control. Upregulated phosphosites are labelled in red and downregulated ones are labelled in blue.

Based on the ATP hydrolase-like activity of LNO, we hypothesized that LNO might affect functional phosphoproteins upon phagocytosis by macrophages. To test this hypothesis, we administered LNO to cells and assessed their ability to dephosphorylate intracellular proteins (Fig. S7†). The intracellular ATP hydrolysis was highly dependent on the concentration of LNO for the macrophages, however, at higher dosage, the ATP level reduced slowly suggesting protein dephosphorylation by LNO was involved at a higher dosage (>5 μg mL^−1^). Based on western blotting, the dephosphorylation of intracellular proteins was evident at higher concentrations of LNO (>5 μg mL^−1^). To better illustrate the double effects of LNO on hydrolysis of phosphate groups, we used different concentrations of LNO for ATP level and protein dephosphorylation studies. We observed that LNO, even at a low dose, induced intracellular protein dephosphorylation. To further understand the impact of LNO cleavage on macrophages, we performed a standard phosphorylation proteomic analysis on RAW 264.7 cells (Fig. 3a–f). Although there is concern about cytotoxicity at a lower dosage of LNO, we calculated that in RAW 264.7 cells the half maximal inhibitory concentration (IC50) of LNO was 8.16 μg mL^−1^. Thus, a dose of 10 μg mL^−1^ was chosen to achieve meaningful results with proper cytotoxicity. It should be noted that after performing quality control of the data, phosphorylation abundance missing in any replicate was removed from further analysis. We observed that phosphorylated proteins changed significantly upon LNO treatment (Fig. 3a and b).

In total, we identified 7503 phosphosites in the control group and 7774 phosphosites in the LNO group with 6857 overlaps (ESI Table S1†). We first examined whether LNO treatment induced omics-scale changes in protein phosphorylation. Based on this criterion, we identified 757 upregulated and 797 downregulated phosphosites (Fig. 3c and Table S2†). These results suggest that LNO treatment alters the phosphorylation levels of a small fraction of proteins.

We next studied whether significant phosphorylation site changes are associated with subcellular localization preferences. The number of up-and downregulated phosphosites was similar in most subcellular locations, including the nucleoplasm, cytoplasm, and endomembrane system (Fig. S8 and Table S3†). Furthermore, we determined the impact of LNO treatment on biological processes and molecular functions. With respect to the upregulated proteins co-involved in the process, we found that upregulated phosphosites were mainly enriched in the regulation of protein catabolic processes, apoptotic pathways, and cellular responses to metal ions, while the downregulated phosphosites were mainly enriched in protein deubiquitination, TOR signalling, and macrophage proliferation (Fig. S9 and Table S4†). Upregulated phosphosites were related to miRNA binding, SUMO transferase activity, histone demethylase activity, and ubiquitin binding functions, whereas downregulated phosphosites were related to snoRNA binding, methyltransferase activity, ubiquitin protein ligase activity, and JUN kinase binding (Fig. S10 and Table S5†). We observed that there were no obvious subcellular localization preferences of LNO due to the shared small coverage of phospho-proteins; however, we discovered that differentially phosphorylated proteins at the same subcellular localization can interact with each other and form a complete network (Fig. S11†).

Finally, we focused on proteins with altered phosphorylation involved in different pathways (Fig. 3d and Table S6†). However, the Pearson correlation coefficient was found to be higher than 0.92 for both protein group and phosphorylation abundance data (Fig. 3e).

Significant differences in phosphosite expression between LNO and control were set at *p*-value < 0.05, and the change in the fold change was larger than 1.5. Importantly, we found that phosphorylation levels of key enzymes in different upstream signalling pathways were altered, especially among which autophagosome formation was regulated through activation of ULK and PI3K complexes. We determined various phosphorylation levels of upstream-activated proteins involved in different pathways (Fig. 3f), including the PI3K-Akt, MAPK, and low-energy pathways.

The presented pathways were partly involved in AMPK, although we did not identify any differences in AMPK phosphorylation. The phosphorylation difference in core kinases, that is, AKT, MAPK2K2, and ERK, resulted in the upregulated phosphorylation of TSC2. With respect to the activated downstream proteins, the phosphorylation level of Deptor, an important inhibitor of the mTOR pathway, was downregulated, which led to sequential activation of ULK and PI3K complexes, resulting in autophagosome formation.

To address whether LNO-induced intracellular dephosphorylation could alter macrophage polarization, we further investigated phospho-proteomics data (Table S7†). It has been reported that Notch activation could suppress SIRPa expression, which promotes M2 macrophage polarization.^29^ While miR-148a-3p promotes M1 and inhibits M2 polarization of macrophages upon Notch activation.^30^ In our phospho-proteome data, phosphorylation levels of Notch pathway proteins were upregulated in five genes (seven sites) and downregulated in one gene (three sites), suggesting that LNO treatment might lead to M1 polarization. The effect of Akt1 kinase was modulated by miR-155 induction and C/EBPβ suppression, which are master regulator genes of M2 differentiation.^31^ We observed that phosphorylation levels of PI3K-Akt pathway proteins were upregulated in 11 genes (16 sites) and downregulated in 11 genes (15 sites). However, the phosphorylation level of AKT1 was significantly upregulated (fold-change 1.85 and *p* value 0.004).

**Fig. 4:**
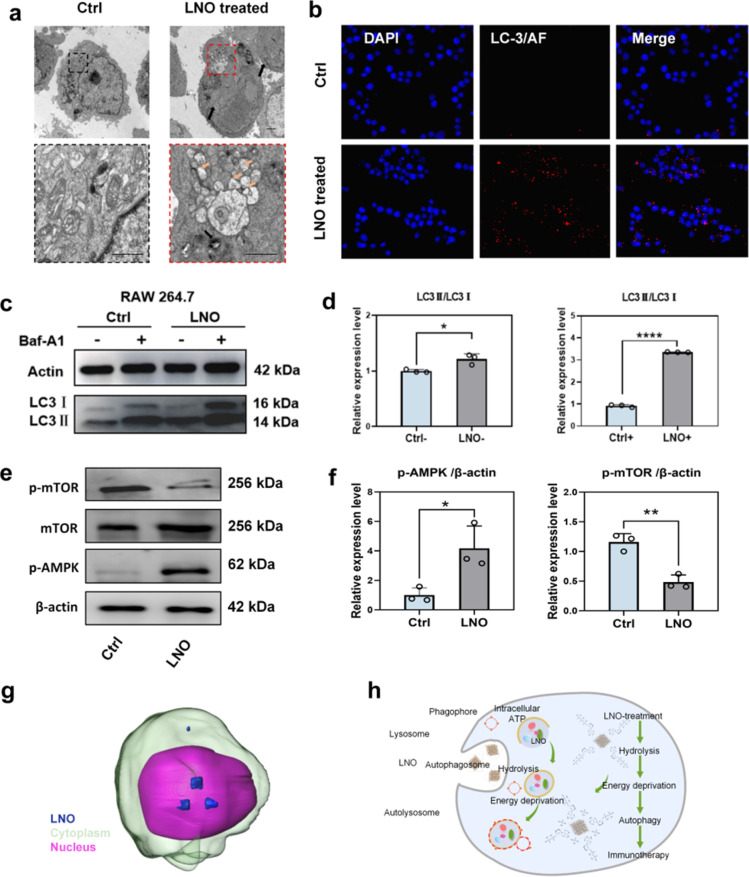
Selective macrophage autophagy mediated by LNO. (a) Subcellular distribution of LNO in RAW264.7 macrophages, non-treated and treated cells as determined by TEM. Black arrow indicates LNO, and orange arrow indicates autophagosome. Scale bar represents 1 μm. (b) Confocal images of LNO-treated RAW264.7 macrophages. Cell nuclei are stained with DAPI (blue), and macrophages are incubated with LC-3 primary antibody and AF633 secondary antibody (red). Scale bar represents 100 μm. Two TEM and confocal microscopy images per sample are obtained to observe cell conditions after LNO phagocytosis. An average of 94.3% of autophagy positive cells are quantified based on confocal images. (c–e) Western blotting analysis of LNO-treated RAW264.7 macrophages. Western blot quantification of LC-3II/LC-3I (d), p-AMPK/β-actin, p-mTOR/β-actin (f) in RAW264.7 macrophages (*n* = 3). (g) Three-dimensional tomographic image showing the accumulation of LNO in RAW264.7 macrophages captured by cryo soft X-ray transmission microscope together with nano-computer tomography (nano-CT). (h) Schematic illustration of ATP hydrolysis by LNO leading to autophagy in macrophages. Statistical significance is calculated by *t* test; *p < 0.5; **p < 0.01; ****p < 0.0001. Data are expressed as mean ± standard deviation for replicated samples (*n* = 3 for (d and f)).

TEM (Fig. 4a) and confocal microscopy (Fig. 4b and S12†) confirmed that LNO effectively activated macrophage autophagy. We observed the accumulation of LNO in macrophages and formation of autophagosomes. Fluorescence microscopic images showed that the red signals (reflecting LC-3 intensities) intensified with increasing doses of LNO, suggesting the occurrence of autophagy in the presence of LNO. Energy deprivation activates autophagy by isolating damaged organelles and cytoplasmic materials from autolysosomes.^8,32^ The dramatic shortage of intracellular ATP and activation of ULK and PI3K complexes were supported by the results of LNO-induced autophagy in macrophages. AMP-activated protein kinase (AMPK) is a central regulator of cellular metabolism, activated by a decrease in intracellular ATP,^33^ and autophagy-associated microtubule-associated protein light chain 3 II (LC-3II) was tested by western blotting (Fig. 4c–f). As a specific inhibitor of autophagy, cells were treated with bafilomycin A1 (Baf-A1), which inhibits autophagic flux. We observed that LC3-II expression increased upon LNO treatment and further increased after Baf-A1 treatment, suggesting an increased synthesis of autophagosomes. These results suggest that LNO activates macrophage autophagy mediated by intracellular ATP hydrolysis through activation of the AMPK/mTOR pathway. We demonstrated that LNO activates the AMPK/mTOR pathway, suggesting its role in inducing autophagy independent of lysosomal degradation, even in the presence of Baf-A1. Additionally, the accumulation of LNO in RAW264.7 cells was visualized using a cryo-soft X-ray transmission microscope together with nano-computer tomography (Nano-CT, Fig. 4g and ESI Movie S1†). Overall, our results suggest that LNO may be a useful nanomaterial in cancer immunotherapy by influencing macrophages (Fig. 4h).

### *In vitro* bone marrow-derived macrophage reprograming by LNO

**Fig. 5:**
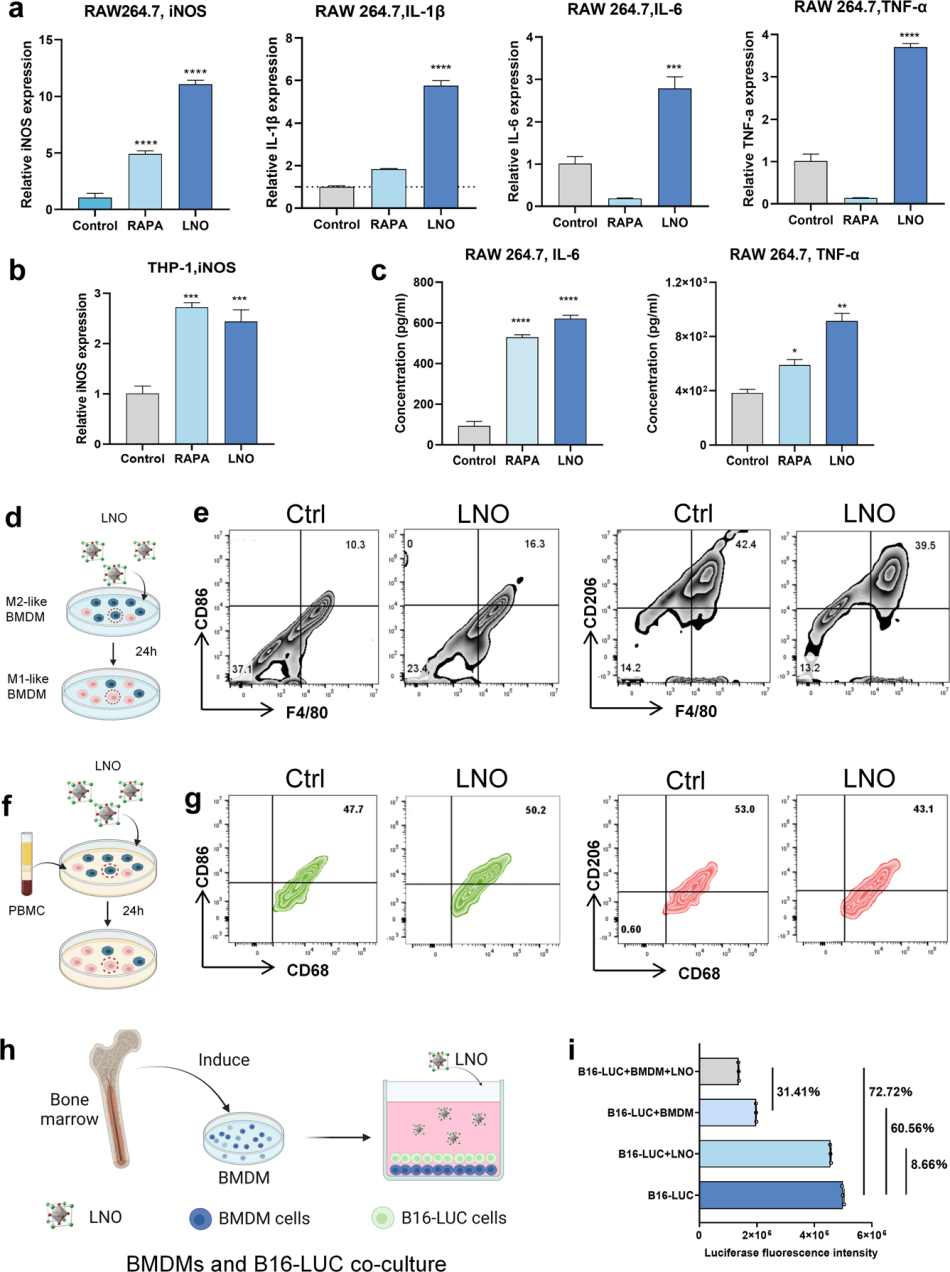
Effective reprogramming of M2-like macrophages *in vitro* and their selective elimination by LNO. (a) Expression of iNOS, IL-1β, IL-6 and TNF-α as measured by qRT-PCR in LNO-treated RAW264.7 macrophages. (b) Expression of iNOS as measured by qRT-PCR in LNO-treated THP-1 macrophages. (c) IL-6 and TNF-α levels in RAW 264.7 cells after LNO treatment. (d) Illustration of repolarization of M2-like BMDMs after LNO treatment for 24 h. (e) Representative flow cytometric analysis of M1-like (CD86^+^) and M2-like (CD206^+^) macrophages gating on CD68^+^ cells. (f) Illustration of repolarization of human mononuclear macrophage after LNO treatment for 24 h. (g) Representative flow cytometric analysis of M1-like (CD86^+^) and M2-like (CD206^+^) macrophages gating on CD68^+^ cells. (h) Schematic illustration of LNO-induced macrophage polarization for the elimination of tumor cells. To evaluate this phenomenon, BMDMs are co-cultured with B16-luciferase cells. (i) Luciferase activity levels after various treatments. Statistical significance is calculated by *t* tests; *p < 0.5; **p < 0.01; ****p < 0.0001. Data are expressed as the mean ± standard deviation for replicated samples (*n* = 3).

*In vitro* studies further showed that LNO significantly expressed iNOS markers in both mouse and human macrophages (Fig. 5a and b) and upregulated pro-inflammatory cytokine expression in macrophages (Fig. 5a and c),^19^ indicating that LNO increases M1-like macrophages.^34^ LNO specifically induced ATP hydrolysis in macrophages and DC (Fig. 1k). We then studied the repolarisation effect of LNO on M2-like bone marrow-derived macrophages (BMDMs, Fig. S13†). Upon treatment with LNO, we observed a notable increase in M1-like (F4/80, CD11b^+^, CD86^+^) BMDMs and a reduction in the M2-like (F4/80, CD11b^+^, CD206^+^) phenotype (Fig. 5d and e). Remarkably, we observed similar effects of LNO on human blood-derived mononuclear macrophages (Fig. 5f, g and S14†). To investigate the ability of LNO-treated macrophages to eliminate tumor cells, we conducted co-culture experiments using luciferase-labelled B16 cells (B16-luc) and BMDM cells. We observed that LNO exhibited the strongest cytotoxic effect against B16 cells in the presence of BMDMs (Fig. 5h and i). We selected B16-LUC as a cell model to evaluate the viability of B16 cells by the fluorescence intensity of luciferase. We observed that LNO could inhibit B16 cells by reprogramming BMDM cells. In the LNO treatment group, the activity of B16-LUC cells decreased by 8.66%, while the activity of B16-LUC cells decreased by 72.72% when BMDM and LNO were added simultaneously. To exclude the effect of BMDM cells on B16 activity, we compared the LNO + BMDM and BMDM treatment groups, and the addition of LNO reduced B16 activity by 31.41%. Taken together, our results show that reprogrammed macrophages play a crucial role in tumor elimination.

### *In vivo* tumor-associated macrophages reprogramming for cancer immunotherapy

**Fig. 6:**
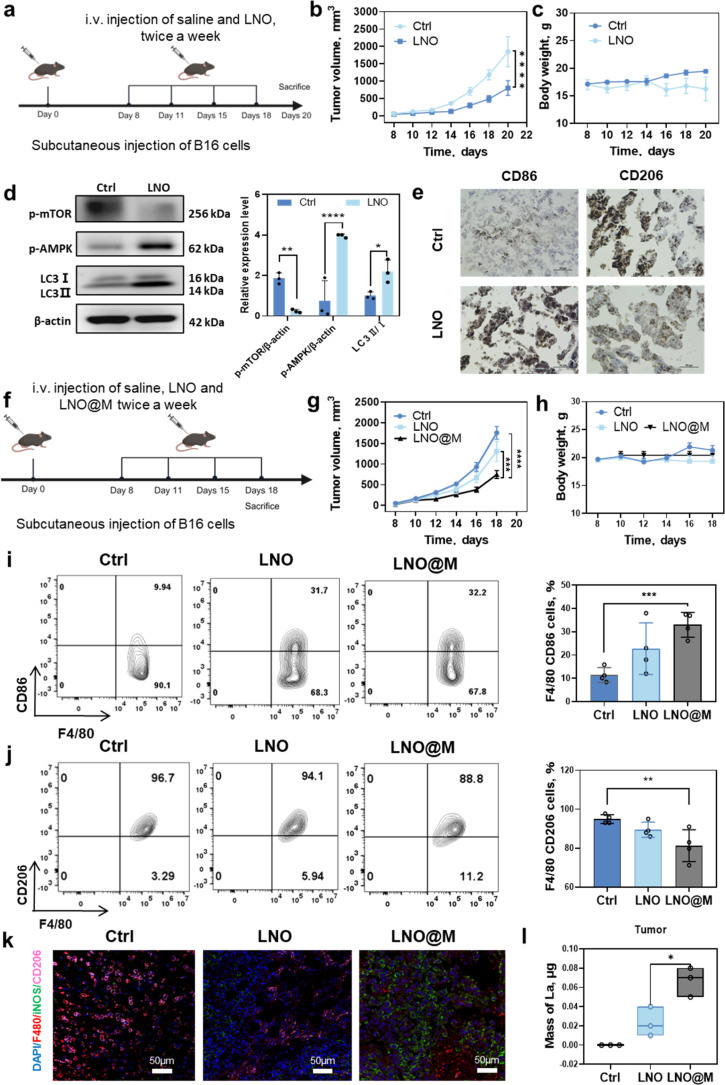
LNO as a cancer immunotherapy agent in a tumor-bearing mouse model. (a) Schematic illustration of *in vivo* antitumor therapy. Tumor cells are subcutaneously injected into C57BL/6 mice and treated with an intravenously injected control (saline) and LNO. (b) Tumor volume, (c) body weight and (d) western blotting analysis of LNO-treated tumor tissues. (e) Tumor immunohistochemistry before and after LNO treatment. (f) Schematic illustration of *in vivo* antitumor therapy by LNO and LNO@M. (g) Tumor volume and (h) body weight of control and treated mice. (i and j) Flow cytometric quantification of cells in B16 tumor-bearing C57BL/6 mice. Flow cytometry analysis and quantification are performed for M1-like (d and f) and M2-like macrophages (e and g) populations in B16 tumors on day 18-post implantation. (k) The immunofluorescence of F4/80 (macrophages, red), iNOS (M1-like macrophages, green), and CD206^+^ (M2-like macrophages, pink) in tumor tissues after various treatments. The scale bar represents 50 μm. (l) The amount of lanthanum in the tumors of B16 tumor-bearing C57BL/6 mice at different treatments. Statistical significance is calculated by one-way ANOVA with Tukey's multiple comparisons test; **p < 0.01; ***p < 0.001; ****p < 0.0001. Data are expressed as mean ± standard deviation for replicated samples (*n* = 3 for (d), and *n* = 4 for (b), (c) and (g–l)).

We investigated the potential of LNO to inhibit *in vivo* tumor growth by regulating tumor-associated macrophages in murine melanoma cell (B16F10)-bearing mice^35^ (Fig. 6a). High dose of LNO (25 mg kg^−1^) were intravenously injected into mice revealing a slight reduction in tumor growth due to the treatment (Fig. 6b and c). Western blotting analysis of tumor tissue indicated that LNO mediated macrophage autophagy through the activation of AMPK/mTOR pathway (Fig. 6d). While Fig. 6d demonstrates the promotion of tissue autophagy, additional experiments (Fig. 1l, 4a–c, and S23†) provide substantial evidence supporting our conclusion that LNO affects macrophage autophagy, with approximately 30–50% of the tumor composition consisting of macrophages.^36^ Furthermore, to determine the effects of LNO on the macrophages *in vitro*, we examined phagocytosis in various cell types (Fig. S15 and S16†). We found that macrophages exhibited lower phagocytosis of LNO compared to cancer cells, but higher than most of normal cell lines (HUVEC and 16HBE). Despite lower phagocytosis compared to cancer cells, LNO demonstrated significant cytotoxicity in macrophages (Fig. 1l), suggesting a stronger effect of LNO on macrophages. In LNO-treated tumor mice, we observed a significant increase in M1-like (F4/80, CD86^+^) and a decrease in M2-like (F4/80, CD206^+^) macrophages, indicating that LNO treatment promotes the reprogramming of TAMs (Fig. 6e).

To enhance tumor targeting, we performed camouflage^37^ using myeloid cell membranes on the surface of LNO (Fig. S17 and Table S8†), which resulted in reduced reticuloendothelial system (RES) uptake and enhanced tumor microenvironment accumulation and suppression (Fig. 6f–h).

To confirm the retention of catalytic activity in LNO@M, we measured intracellular ATP levels in RAW264.7, demonstrating that LNO@M maintained its efficient capability for ATP hydrolysis even after being coated with cell membranes (Fig. S18†). Next, we investigated the effects of different LNO formulations on the function of TAMs (Fig. 6i–k and S19†). The injection of 150 μg of LNO and LNO@M into B16 tumor-bearing mice revealed that 0.02% of LNO and 0.07% of LNO@M reached tumor after 24 h of treatment. Interestingly, we found that LNO@M significantly increased the proportion of M1-like macrophages and inhibited M2-like macrophages and dendric cells compared with bare LNO. There were no significant changes in other immune cells, suggesting that LNO specificity induces macrophage reprogramming. A possible reason is that macrophages are the first line of defense of the body's immunity and can recognize extraneous pathogens (Fig. S20†). ICP-MS results also showed that, compared with LNO, LNO@M enveloped with the myeloid cell membrane accumulated more in tumors and less in other tissues. We injected 150 μg of LNO and LNO@M into B16 tumor-bearing mice, 0.02% of LNO and 0.07% of LNO@M (Fig. 6l) reached the tumor after 24 h of treatment. By comparing bare LNO with LNO@M, there was a significant difference in their accumulation in various tissues, such as 2 folds and 1.5 folds reduced accumulation of LNO@M in liver, kidney, and lung tissues (Fig. S21†), minimizing the accumulation of nanoparticles in these organs. However, the LNO@M formulation showed a remarkably higher accumulation of approximately 3.5 folds in tumor tissue (Fig. 6l) than bare LNO, suggesting preferential accumulation in tumor cells, which is vital in cancer treatment strategies.

To further verify the biosafety and therapeutic efficiency of LNO and LNO@M, the main organs of the mice in each group were harvested for H&E staining. As shown in Fig. S22,† no obvious histopathological lesions were observed in the organs of any group, indicating good biosafety of LNO@M. To confirm the selective effects of LNO on macrophages *in vivo*, we studied the cell viability of cancer cells (B16) after treatment with different concentrations of LNO. We observed that LNO induced much lower toxicity than LNO-treated macrophages (Fig. S23†). Furthermore, we studied the dephosphorylation (Fig. S24†) and ATP hydrolysis (Fig. S25†) of LNO *in vivo*. We observed that 31% of the tumor tissue proteins were dephosphorylated and 40% ATP was hydrolyzed upon LNO treatment. Overall, these results revealed that LNO could significantly change the M1/M2 ratio in the TME, thus acting as a promising nanomedicine for cancer immunotherapy.

## Conclusions

TAMs can be interconverted with changes in the tumor microenvironment and play an important role in the stages of tumorigenesis, progression, invasion, and metastasis; their typing and number may also be used as indicators of tumor prognosis. In tumor-bearing mouse models, macrophages promote cancer initiation and malignant progression by stimulating angiogenesis, increasing tumor cell migration, and suppressing antitumor immunity. Tumor-associated macrophages (TAMs) are promising targets for cancer immunotherapy through ablation or differentiation of macrophages. Nanoparticle-based cancer immunotherapy has shown promising therapeutic potential in clinics, with the majority of studies focusing on the use of nanoparticles as drug delivery agents and for the direct treatment of cancer cells. Although several nanoparticles have shown immune-regulatory responses, there is a lack of development of selective therapies that effectively reprogram macrophages in tumors. Herein, we first proposed a conceptional TAM-targeting strategy by coating the myeloid cell membrane on LaNiO3 (LNO) nanomaterial and show that the employed LNO acts as a phosphatase-like nanomaterial with efficient catalysis of intracellular ATP hydrolysis and protein dephosphorylation at the interfaces (Fig. 1 and 2). LNO causes energy deprivation in macrophages and efficiently induces autophagy, even at low dosages. We revealed that nickel and oxygen are the active sites of LNO, determining the catalytic activity resulting in ATP hydrolysis (Fig. 3). Moreover, we observed that LNO, even at a low dose, showed a strong capability of sequestrating phosphate groups, resulting in the de-phosphorylation of intracellular proteins. Both the energy deprivation and upstream protein alterations due to dephosphorylation activated the AMPK/m-TOR pathway induced macrophage autophagy, suggesting that LNO could be used as a promising immunotherapy agent for cancers (Fig. 4 and 5). We studied the repolarization effect of LNO on M2-like bone marrow-derived macrophages and observed that reprogrammed macrophages play a crucial role in tumor elimination (Fig. 6). To enhance the ability of LNO-treated macrophages to eliminate tumor cells, we combined LNO surface coating with myeloid cell membranes, which resulted in higher accumulation of LNO in tumor-associated macrophages and promoted their reprogramming, resulting in significant changes in the M1/M2 ratio, and enhanced tumor elimination *in vitro* and *in vivo* for cancer immunotherapy (Fig. 6). These unique properties of LNO enable its use as a novel reagent in cancer immunotherapy.

In conclusion, we have revealed the potential of LNO-based nanomedicine as a cancer immunotherapeutic reagent by inducing macrophage autophagy *via* catalysis of phosphate group hydrolysis. Our findings offer mechanistic insights into how rare-earth lanthanum nickel oxide nanomaterials promote autophagy that selectively interferes with tumor-associated macrophages and provide a valuable strategy for the development of immune cell-targeted agents for cancer immunotherapy.

## Data availability

The data generated in this study is available with the article, Supplementary information,† and Supplementary video.† The proteomic data generated by mass spectrometry, including raw data and search results have been deposited to the ProteomeXchange Consortium (http://proteomecentral.proteomexchange.org) *via* the PRIDE partner repository with the dataset identifier: PXD051695. The mouse proteomic dataset is available on the UniProt website (https://www.uniprot.org). The bioinformatic analysis results are presented in ESI Tables S1–S7.†

## Author contributions

This project was conceived by D. B., L. W. and Y. L. and designed by D. B. and supervised by L. W., Y. L., X. G., and C. C. LNO was synthesized by X. W. under the supervision of H. W. DFT calculations were conducted by X. G. and J. L. under supervision of X. G. WB experiments, LNO@M synthesis and tumor-bearing mice model experiments were performed by S. L. with the assistance of Q. L. under supervision of G. Z. and Y. L. Phospho-proteomics results were processed by Y. L. and D. B. under supervision of J. W. through continues discussion with D. B., H. D. and L. W. NMR experiments were performed by D. B. with the assistance of F. L. The remaining experiments were performed by D. B. with the assistance of K. L. and R. C. The manuscript was written with the contributions of all authors. All authors approved the final version of the manuscript.

## Conflicts of interest

The authors declare no competing financial interests.

## Supplementary Material

SC-015-D4SC01690D-s001

SC-015-D4SC01690D-s002

SC-015-D4SC01690D-s003
