# Supplementary material for: A phosphatase-like nanomaterial promotes autophagy and reprograms macrophages for cancer immunotherapy
Source: Chem Sci. 2024 Jun 17;15(28):10838–50. doi: 10.1039/d4sc01690d (PMC11253186; doi:10.1039/d4sc01690d)
Supplement: SC-015-D4SC01690D-s001 [file SC-015-D4SC01690D-s001.pdf]

Electronic Supplementary Material (ESI) for Chemical Science.
This journal is © The Royal Society of Chemistry 2024

Supporting Information

A phosphatase-like nanomaterial promotes autophagy and reprograms macrophage for cancer immunotherapy

Didar Baimanov[1,#], Su Li[2,3,#], Xuejiao J. Gao[4,#], Rui Cai[1], Ke Liu[1], Junjie Li[4], Yuchen Liu[5], Yalin Cong[1,9], Xiaoyu Wang[6], Fen Liu[7], Qi Li[2], Guofang Zhang[2], Hui Wei[6], Jian Wang[5], Chunying Chen[1,9], Xingfa Gao[8,*], Yang Li[2,10,*] and Liming Wang[1,*]

[1] CAS Key Laboratory for Biomedical Effects of Nanomaterials and Nanosafety, CAS-HKU Joint Laboratory of Metallomics on Health and Environment, Laboratory of Nano-Bio Interface Science, Institute of High Energy Physics, Chinese Academy of Sciences and National Center for Nanoscience and Technology of China, Beijing 100049, P. R. China

[2] Laboratory of Immunology and Nanomedicine & China-Italy Joint Laboratory of Pharmacobiotechnology for Medical Immunomodulation, Laboratory of Inflammation and Vaccines, Shenzhen Institute of Advanced Technology, Chinese Academy of Sciences, Shenzhen 518055, P.R. China

[3] Division of Allergy & Immunology, Department of Biosciences & Medical Biology, Paris Lodron University of Salzburg, 5020 Salzburg, Austria

[4] College of Chemistry and Chemical Engineering, Jiangxi Normal University, Nanchang 330022, P. R. China

[5] State Key Laboratory of Medical Proteomics, Beijing Proteome Research Center, National Center for Protein Sciences (Beijing), Beijing Institute of Lifeomics, Beijing 102206, P. R. China

[6] College of Engineering and Applied Sciences, Nanjing National Laboratory of Microstructures, Jiangsu Key Laboratory of Artificial Functional Materials, Nanjing University, Nanjing 210093, P. R. China

[7] State Key Laboratory of Natural and Biomimetic Drugs, School of Pharmaceutical Sciences, Peking University, Beijing 100191, P. R. China

[8] Laboratory of Theoretical and Computational Nanoscience, National Center for Nanoscience and Technology of China, Beijing 100190, P. R. China

[9] New Cornerstone Science Laboratory, National Center for Nanoscience and Technology of China, Beijing 100049, P. R. China

[10] The Key Laboratory of Biomedical Imaging Science and System，Chinese Academy of Sciences, Shenzhen, P. R. China

[#] These authors contributed equally.

* To whom correspondence should be addressed: wangliming@ihep.ac.cn, yang.li@siat.ac.cn, gaoxf@nanoctr.cn

This PDF file includes:

Materials and Methods
Figures and Tables
Legends for Movies S1
Legends for Datasets S1
SI References

Other supporting materials for this manuscript include the following:

Movies S1
Datasets S1

Materials and methods

Chemicals and Reagents. Adenosine triphosphate (ATP), adenosine diphosphate (ADP), adenosine monophosphate (AMP), guanosine triphosphate (GTP), guanosine diphosphate (GDP), guanosine monophosphate (GMP), 4-(2-hydroxyethyl) piperazine-1-ethanesulfonic acid (HEPES) were all supplied by Shanghai Macklin Biochemical Co., Ltd. Dulbecco's Modified Eagle Medium (DMEM), RPMI-1640, phosphate buffered saline (PBS) were obtained from Wisent Inc. Fetal bovine serum (FBS) was purchased from Biological Industries. CCK-8 assay (cat.# CK04-1000T), LDH release (cat.# CK12-2000T) were obtained from Dojindo. ATP kit (cat.# S0027) and SDS-PAGE preparation kit were obtained from Boyetime (China). DAPI (4′,6-diamidino-2-phenylindole) was purchased from Molecular Probes. Anti-Rabbit LC3 antibody (cat.# 12741), anti-Rabbit β-actin antibody (cat.# 4967), anti-Rabbit phospho-AMPK antibody (cat.# 2535) and anti-Rabbit phospho-m-TOR antibody (cat.# 5536) antibodies and Bafilomycin A1 were obtained from Cell Signaling Ltd. Secondary antibodies (HRP and Alexa Flour 633) were obtained from Gene-Protein Link Biotech. Immobilon-P transfer membranes were purchased from Millipore. RIPA lysis buffer (Pierce RIPA Buffer), Pierce bicinchoninic acid (BCA, cat.# 23225) protein assay was from Thermo Scientific™. Phosphatase inhibitor (PhosSTOP) and protease inhibitor (cOmplete mini EDTA-free) tablets were purchased from Roche. Recombinant Murine M-CSF (cat.# 315-03). and Recombinant Murine IL-4 (ca.t# 214-14) were purchased from PeproTech (USA). Luciferase Assay System (cat.# E1910) was purchased from Promega (USA). Pro-Q Diamond phosphoproteins (cat.# P33301) and SYPRO Ruby protein gel stains (cat.# S12001) were purchased from Invitrogen. Malachite Green Phosphate Assay Kit (cat.# MAK307) was purchased from Sigma-Aldrich. Anti-mouse $CD16/32^+$ antibody (cat.# 101301), FITC anti-mouse $CD45^+$ antibody (cat.# 157607), APC anti-mouse $CD11b^+$ antibody (cat.# 101211), Alexa Fluor® 700 anti-mouse $Ly\text{-}6C^+$ antibody (cat.# 128023), PerCP anti-mouse $Ly\text{-}6G^+$ antibody (cat.# 127653), APC anti-mouse $CD3^+$ antibody (cat.# 100235), PE anti-mouse $CD8a^+$ antibody (cat.# 100707), PE anti-mouse $CD11c^+$ antibody (cat.# 117307), PE anti-mouse F4/80 antibody (cat.# 111603), APC/Cyanine7 anti-mouse $CD86^+$ antibody (cat.# 105029), PE/Cyanine5 anti-mouse $CD206^+$ antibody (cat.# 141739), were purchased from Biolegend. Human $CD14^+$ MicroBeads (cat.# 130-050-201) were purchased from Miltenyi Biotec (Germany). FITC anti-human $CD68^+$ antibody (cat.# 333805), APC anti-human $CD86^+$ antibody (cat.# 374207), APC/Cyanine7 anti-human $CD206^+$ antibody (cat.# 321119), were purchased from Biolegend. All chemicals and agents were used without further purification unless otherwise noted. The applied water (18.1 MΩ·cm-1) in the experiments was purified from a Milli-Q Ultrapure Water Systems.

Characterization of LNO. LNO was synthesized as previously described[1]. LNO was dispersed onto a holey carbon film on copper grids and then observed under a TEM (Tecnai G2 20 S-TWIN) from Field Electron and Ion Co. (FEI, USA) at an acceleration voltage of 200 kV. The surface charge was determined by a Zetasizer Nano ZS (Malvern) spectrometer. XPS analysis was carried out using ESCALAB250Xi. The survey spectra are an average of 10 scans taken with a pass energy of 200.00 eV and a step size of 1 eV.

Evaluation of pH-dependent ATP hydrolysis. To determine the optimal conditions for ATP hydrolysis, the ATP solution was incubated with LNO (50 μg/mL) at different pH (4.5, 5.5, 7.4) by ATP level determination kit. ATP was dissolved in HEPES buffer and adjusted pH by NaOH (50 mM). The relative catalytic activity of LNO was compared to ATP at (10 μM, pH 7.4, 37°C).

Malachite Green Assay. The hydrolyses of ATP and ADP were measured by the detection of phosphate product using a malachite green assay in 96-well microtiter plates. Assays were performed in 100 μL HEPES buffer solution with ATP (10 μM) or ADP (10 μM) and then were started with the addition of 100 μL LNO or apyrase to the above solution. Final reaction concentration of LNO (50, 25, 12.5, and 6.25 μg/mL) and apyrase (20 mM). The reactions were run for up to 30 min at a constant temperature shaker (37°C, 150 rpm). All samples were performed in triplicates and standardized against a blank. The reactions were terminated using the malachite solution, the absorbance was measured at 620 nm in a microplate reader.

The selective hydrolysis of phosphate-containing molecules. The catalytic activities toward various phosphate molecules were measured using the above malachite green assay. The concentrations of molecules including ATP, adenosine monophosphate (AMP), guanosine triphosphate (GTP), guanosine diphosphate (GDP), and guanosine monophosphate (GMP), were set as 10 μM. The reactions were run for up to 30 min at a constant temperature shaker (37°C, 150 rpm) using 50 μg/mL of LNO. The relative hydrolysis activity of LNO toward ATP and GTP are presented as 100%. The catalytic activities of $La(NO_3)_3$, La_2O_3, and NiO aqueous solutions toward ATP hydrolysis were carried out. The hydrolysis efficiency toward ATP (10 μM) was measured using 50 μg/mL metal ions diluted in ultrapure water. The relative catalytic activity of metal ions was compared to ATP at (10 μM, pH 7.4, 37°C), which was set as 100%.

Enzyme-like activity of LNO. The cleavage/hydrolysis rate of ATP after interaction with LNO was determined by a malachite green assay kit. In detail, ATP was set at 265.6, 132.8, 66.4, 33.2, 16.6, 8.3, 4.15, and 2.08 μM prior to interaction with LNO (50 μg/mL) for 10 min (pH 4.5, 37°C).

Ten minutes were chosen based on the cleavage rate. The Michaelis constant (Km) and maximum reaction rate (Vmax) were calculated by the Michaelis-Menten equation.

^{31}P-NMR spectra. The hydrolysis of ATP and ADP by LNO and apyrase enzyme were tracked and characterized by ^{31}P-NMR. In detail, ATP (0.1 mmol) or ADP (0.1 mmol) were dissolved in HEPES solution (pH 4.5, 0.12 mmol). Further LNO (50 μg/mL) or apyrase (3 μg/mL) were added into solution and incubated at 37°C for 30 min, followed by filtration of LNO and heat-inactivation for an enzyme (95°C for 10 min). 10% (v/v) D_2O was added into each sample solution prior to ^{31}P-NMR measurement. Furthermore, the conversion rate of ATP and ADP could be analyzed based on β-ATP peak (-20.8 ppm), AMP peak (3.2 ppm), α-ATP, and α-ADP peaks (10.7 ppm).

DFT simulation. The (001) slabs of LNO with 6 atom layers were constructed and optimized with full atoms. The G-type antiferromagnetic (G-AFM) state was applied during optimization since it is reported as the ground state by the previous study[1]. During the optimization of the intermediates, the bottom two atomic layers are fixed to mimic the bulk material. The generalized gradient approximation with the Perdew–Burke–Ernzerhof functional with vdW corrections estimated in the DFT-D3BJ form[2-4] was used for all geometry optimizations and energy calculations. An energy cut-off of 450 eV and Gaussian smearing of 0.05 eV were utilized. The Hubbard U correction with U defined as U_{eff} was set as 6.2 for Ni to treat the strong on-site Coulomb interaction of the localized d electrons[1]. For all calculations, the (3 × 3 × 1) Monkhorst−Pack meshes were used for the k-point samplings[5]. The convergence thresholds for the electronic structure and forces were set to be 10^{-4} eV and 0.05 eV $Å^{-1}$, respectively. All calculations were performed using the VASP package with projector-augmented wave pseudopotential[6-8].

Cell culture. RAW264.7 and J774A.1 cells used for *in vitro* experiments were purchased from Procell Life Science & Technology Co., Ltd. DC2.4, HUVEC and 16HBE cells were purchased from ATCC. RAW264.7 and J774A.1, HUVEC cells were maintained in DMEM supplemented with 1% penicillin−streptomycin supplied with 10% FBS. DC2.4, 16HBE, Jurkat T, B16F10 and luciferase labeled B16 (B16-luc) cells which was established by Laboratory of Inflammation and Vaccines, Shenzhen Institute of Advanced Technology were cultured in RPMI-1640 supplemented with 10% FBS, and 1% penicillin−streptomycin. For Jurkat T and THP-1 cells, cell culture medium was additionally supplied with 0.05mM β-mercaptoethanol. To obtain macrophages from THP-1 cells, cells were introduced to 10 mg/mL PMA for 48 h. Obtained differentiated macrophages were used to test macrophages polarization ability by RT-qPCR.

Cytotoxicity assays. RAW246.7, J774A.1, HUVEC, 16HBE and B16F10 cells were seeded at a density of 5000 cells per well, Jurkat T cells and DC2.4 were seeded at cells density of 8000 cells per well in 96-well plates and incubated for 24 h. Cell culture medium containing various concentrations of LNO (0–80 μg/mL) was subsequently added to the cells in the respective wells and further incubated for another 24h. Cytotoxicity was measured by CCK-8 assay and LDH assay kits (for details see manufacture protocol). Cellular ATP level was determined by luminescence record (see manufacture protocol for details). To ensure the quality of obtained results, each sample was normalized based on the protein quantity (determined by BCA assay kit) before measurement.

Cryo-soft X-ray transmission microscope-based Nano-CT imaging. Single cell imaging was performed by cryo-soft X-ray transmission microscope (TXM, nano-CT) at the beamline BL07W of the National Synchrotron Radiation Laboratory (NSRL, Hefei, China) to observe the accumulation of LNO (10 μg/mL) within RAW264.7 macrophages. 24 h post-treated macrophages were placed on the non-carbon formvar of a copper 100 mesh after cell fixation with 10% paraformaldehyde for 20 min, followed by cell dehydration and liquid nitrogen immersion. The soft X-ray520 eV energy focused onto the cell and magnified by a CCD camera obtaining 30 nm spatial resolution. Cells were rotated from -60° to +60°, and a series of projected images were collected 121 times in sequence, with 1-degree intervals and 2-s exposure time. 3D tomographic reconstruction was performed via XMReconstruction by aligning the tilt series using XMController, and cell structure was visualized via Amira (FEI Company). To visualize cellular components and LNO, we labeled the cytoplasm in magenta, LNO in blue, and the nucleus in cyan.

Confocal microscopy. Prior to experiments, 35-mm confocal dishes were treated with cell adherent reagent (1:200× dilution, Applygen Technologies Inc.) for 30 min at 37°C and were washed with PBS. RAW246.7 and J774A.1 (3×10^5) cells were cultured with 35-mm dishes and treated with LNO (5 and 10 μg/mL) for 24 h. Cells were fixed with 4% formaldehyde (15 min, RT), and after triplicated wash (PBS), cells were incubated with 0.1% Triton X-100 supplied PBS (10 min, RT). Followed by blocking by 1% BSA dissolved in PBST for 30 min. Then cells were incubated with LC3 antibody (1:100× dilution) for 1 h at 4°C. After being washed three times with PBS (10 min), secondary antibody AF633 (1:500 × dilution) for 1 h at RT. Finally, the cell nuclei were labeled with DAPI.

Western blotting. After incubation with various concentrations of LNO (0-80 μg/mL) for 24 h, RAW246.7 were collected by centrifugation at 1200 rpm for 3 min and lysed with RIPA buffer supplied with protease inhibitor (100 μL). Subsequently, lysed cells were centrifuged at 12,000 rpm

for 10 min at 4°C to afford clear lysate. The protein content of the cell lysate was determined using the BCA protein assay kit. All samples were diluted with 6× Loading Buffer and then boiled for 5 min (95°C). The samples were separated by polyacrylamide gel electrophoresis and transferred to a PVDF membrane. Next, the membrane was incubated in a blocking buffer for 30 min and treated overnight with antibodies (1:1000× dilution) at 4°C under continuous stirring. After being washed with Tris-buffered saline (TBST: 0.05% Tween-20) three times, the membrane was further incubated with a secondary antibody (1:3000 dilution) for 1 h at RT. After triplicated wash with TBST buffer, the PVDF membrane was visualized.

Preparation of bone marrow-derived macrophages (BMDMs). To isolate non-activated mouse BMDMs, bone marrow was collected from the femurs and tibiae of female C57BL/6 mice according to the previous reports[9]. Briefly, femurs and tibias were flushed with PBS and removed from the muscle tissue. Bone marrow cells were obtained by flushing the bones with PBS and filtered through a 70 μm cell strainer. The cells differentiated into macrophages with an incubation period of 7 days in a complete RPMI-1640 medium. The medium was half renewed every two days and fully changed at the fifth day. BMDMs were stimulated with 20 ng/mL macrophage colony-stimulating factor (M-CSF) and 20 ng/mL Interleukin-4 (IL-4) for 24 h to obtain M2-like macrophages. On day 7, the loosely adherent unmatured BMDMs were gently harvested for further use.

Preparation of human mononuclear macrophages. Human blood was collected from healthy donors at the Shenzhen Blood Center with their informed consent with approval of the ethics committee (SIAT-IRB-230415-H0648) and complied with all relevant ethical regulations. The gender difference and sex specific analysis were not considered in this study. Peripheral blood mononuclear cells (PBMCs) from each sample were isolated using Ficoll-Paque™ PLUS Media (GE Healthcare, Chicago, IL, USA) within 2 h, according to the manufacturer's instructions. Briefly, 10 mL of whole blood was transferred from the collection vessel to a 50 mL EP tube; an equal volume of PBS solution was added to the EP tube to dilute the blood. Then, the diluted blood was added to 15 mL of Ficoll and centrifuged at 500×*g* for 20 min (acceleration 3, deceleration 0); next, the buffy coat was carefully transferred to a new tube and diluted with PBS to a total volume of 20 mL. The samples were centrifuged again at 600× *g* for 6 min, after which the buffer was discarded, and the cell pellet was resuspended in 3 mL PBS. After incubation of PBMC with MACS Microbeads conjugated with mouse monoclonal antibody raised against human $CD14^+$ protein, $CD14^+$ cells were recovered by washing PBMC-labelled cells in PBS. $CD14^+$ cells were eluted in

PBS after magnetic detachment of the column. Then, cells were resuspended in cell culture medium to study the repolarization effects mediated by LNO.

Co-culture of BMDMs with B16-luciferase. After obtain the BMDM cells following the method mentioned before, we seed the BMDM cells in 6-well plate ($5x10^4$ per well), then add $5x10^4$ per well B16-LUC cells and 2.5 μg/ml LNO. After 24h, cells were then washed by warm DMEM to remove extracellular LNO. Then cells were lysed with 50 μL lysis buffer (E1910; Promega, Madison, WI, USA) and shaking for 5 min at RT. The cell lysate (30 μL) was mixed with 20 μL luciferase substrate, and the luciferase activity was immediately measured with a microporous plate luminescence detector (Glomax 96, Promega) and expressed as relative luciferase units (RLU). Experiments were run in triplicate, and the RLU were S7analyzed for each biological sample with three technical replicates.

Synthesis and characterization of LNO@M. Mouse bone marrow was initially harvested to obtain myeloid cell membranes (M). Harvested cells were digested and the resulting cell precipitate was mixed with cell lysis buffer containing 1% PMSF protease inhibitor, followed by incubation on ice for 15 minutes. The cells were then completely lysed through repetitive freeze-thaw cycles. The lysed cell mixture was subsequently centrifuged at 700 g for 10 minutes at 4 °C. The obtained supernatant was further centrifuged at 14,000 g for 30 minutes at 4 °C to isolate the myeloid cell membranes (M). The M was resuspended in phosphate buffered saline (PBS) and stored at 4°C for later use.

To prepare LNO@M, LNO (200 μg) was dispersed in 1 mL of the M suspension obtained from 5×10^6 myeloid cells. The dispersion was then extruded 11 times using an Avanti mini extruder equipped with a 400 nm pore polycarbonate membrane (Avanti Polar Lipids, Inc., Alabaster, AL). Subsequently, the dispersion was centrifuged at 10000 g for 6 minutes at 4°C to obtain the LNO@M. Transmission electron microscopy (TEM) imaging was performed using a JEM-1400plus analytical electron microscopy to observe the sizes and morphologies of LNO@M[10]. Hydrodynamic sizes and zeta potentials were measured using a Malvern Zetasizer Nano ZS model ZEN3600 (Worcestershire, UK). The presence of membrane proteins on the surface of M and LNO@M was examined using SDS-polyacrylamide gel electrophoresis (SDS-PAGE) assays.

***In vivo* anti-tumor activity of LNO.** The murine melanoma cell line B16F10 was purchased from ATCC and cultured with DMEM containing 10% FBS at 37 °C in a humidified atmosphere with 5% CO_2. C57BL/6 female mice were purchased from Vital River Laboratories (Beijing, China).

Animal experiments were performed in accord with the recommendations detailed in the Guidelines for Care and Use of Laboratory Animals of the Shenzhen Institute of Advanced Technology, CAS, Shenzhen, with approval ID SIAT-IACUC-230323-YYS-LY-A2257. Three mice per group were employed in the following experiments. B16F10 cells (2.5×10^5) were subcutaneously injected into the flank of 6-week-old female C57BL/6 mice. When the tumor volume reached about 100 mm^3, the mice were randomly divided into two groups. The mice were *i.v.* injected with saline and LNO at the dosage of 500 μg per mouse (20 g) in total four times every 3 days. To enhance the tumor delivery of the LNO, perovskites were coated myeloid cell membranes LNO@M. Membrane coated LNO perovskites were characterized prior to animal experiments. The mice were *i.v.* injected with saline, LNO and LNO@M at the dosage of 150 μg per mouse (20 g) in total four times every 3 days. Tumors were measured every 2 days with a caliper, and the volume (length × (width) 2/2) was calculated. Animals were euthanized if they exhibited signs of distress or when the total tumor volume reached 2000 mm^3. The tumors, spleens, and draining lymph nodes were collected and subjected to further analysis.

Flow cytometry. The immune cells in the tumor were collected by digesting tumor tissues. Tumor tissues were cut into pieces and transferred to hanks buffer containing collagenase IV (0.3 mg/mL), hyaluronidase (100 μg/mL), and DNase I (50 μg/mL) for 120 min shaken at 37°C. Cells were filtrated through a 40 μm cell strainer and further purified based on the density gradient method. Before incubation with a specific fluorescence antibody, an anti-$CD16/32^+$ antibody was used as the FC-blocking antibody to prevent nonspecific binding. Tumor-associated macrophages (TAMs) ($CD11b^+$, $F4/80^+$), M1-like (F4/80, $CD86^+$) and M2-like (F4/80 $CD206^+$) macrophages, DC (MHC II, $CD11b^+$), T ($CD3^+$), $CD8^+$ ($CD3^+$, $CD8^+$) cells and neutrophils ($Ly6C^{int}/Ly6G^+$) were evaluated by flow cytometry.

Immunohistochemistry. Tissue samples of various scaffolds were incised along the major axis and embedded in paraffin. Serial sections (3–5 μm) were used for immunohistochemistry staining. Olympus microscope BX51 and digital camera DP70 (Olympus, Tokyo, Japan) was used to photograph. Histological evaluation of all sections was performed by two independent examiners.

Quantitative real-time PCR. Total RNA was isolated from cells using TRIzol (Thermo Fisher Scientific). For qRT-PCR analyses of mRNAs, first-strand cDNA was synthesized. The expression levels of target genes were examined with specific primers listed in the figure legend. Primers of mouse are presented:

Primer	Forward	Reverse
m-IL-1β	TGAACTCAACTGTGAAATGCCAC	CAGGTATTTTGTCGTTGCTTGGT
m-iNOS	GGAGTGACGGCAAACATGACT	TCGATGCACAACTGGGTGAAC
h-iNOS	CTATTCCCAGCCCAACAACAC	GATGGAGTCACATGCAGCTTG
m-IL	AACCAAGAGATAAGCTGGAGTCAC	AACGCACTAGGTTTGCCGAG
m-TNF-α	GACGTGGAACTGGCAGAAGAG	TTGGTGGTTTGTGAGTGTGAG

In vitro cytokine concentrations analysis. The cytokine concentrations of mouse IL-6 (R&D Catalog #: DY406) and TNF-α (R&D Catalog #: DY410) were determined from the supernatant of RAW264.7 treated with LNO. All experiments were performed using corresponding ELISA Kits according to the manufacturer′s instructions.

Intracellular phospho-protein level. LNO (10 μg/mL) treated (24 h) RAW264.7 cells were lysed, reduced, and alkylated in RIPA lysis buffer (Pierce RIPA Buffer, Thermo Scientific™) supplemented with one tablet of phosphatase inhibitor (PhosSTOP, Roche) and protease inhibitor (cOmplete mini EDTA-free, Roche) per 10 mL buffer. Cells were sonicated with a sonic probe (Biosafer) for 15 cycles of 30 s, centrifuged at 12000 rpm for 10 min at 4°C to afford clear lysate, and finally diluted in standard 1X loading buffer. A BCA protein assay was performed to quantify the protein amount. Proteins were delipidated and desalted prior to the electrophoresis experiment as described in the Pro-Q diamond phosphoprotein gel staining protocol.

LC-MS/MS. Cells were dissolved in 300 μL 10% of the lysate supplied with the protease inhibitor were used to collect proteins by centrifugation. Proteins were trypsin digested into peptides and extracted from gel for label-free LC-MS/MS analysis by Beijing Qinglian Biotech Co., Ltd. For LC–MS/MS analysis, Nanoflow LC-MS/MS analysis of tryptic peptides was conducted on a quadrupole Orbitrap mass spectrometer (Q Exactive HF-X, Thermo Fisher) coupled to an EASY nLC 1200 ultrahigh pressure system (Thermo Fisher) *via* a nano-electrospray ion source[11]. Mass spectrometry was operated using data-dependent acquisition mode. Positive ion spray voltage was set to 2,100 V, with the ion transfer tube temperature at 320 °C. The detector was set to mass spectrometry full scanning range of m/z 350-1500, primary mass spectral resolution set to 120,000 (200 m/z), AGC to 3×10^6, C-trap maximum injection time of 80 ms. Mobile phase A consisted of 100% water, 0.1% formic acid and B liquid consisted of 80% acetonitrile, 0.1% formic acid. The most intense ions from the full scan were isolated with an isolation width of 1.6 m/z. Following higher-energy collisional dissociation (HCD) with a normalized collision energy (NCE) of 27, MS/MS spectra were collected in the Orbitrap (15,000 resolution) with an AGC target of 5E4. Percolator was used to filter peptide spectral matches and peptides to a false discovery rate (FDR)

of less than 1%. In this study, porous agarose TiO_2 beads with paramagnetic properties were used as a tool to enrich phosphor-peptides in model and complex samples. MS/MS spectra from each LC-MS/MS run were searched against the mouse database at uniprot website (2023.03.07) using the software Proteome Discoverer (Thermo Fisher, v. 2.4). The mass spectrometry proteomics data have been deposited to the ProteomeXchange Consortium via the PRIDE[12] partner repository with the dataset identifier PXD051695.

Bioinformatic analysis. To obtain a high confidence of phosphorylated site, data were processed by strict quality control process. Pearson correlation coefficient of each protein and phosphorylated site was calculated based on original intensities. Correlation within group was above 0.99, indicating a good data quality. The unadjusted p-value was used for differential phosphorylated proteome analysis. After data mean normalization, phosphorylated site missing in any replicate was removed. The total number of phosphorylated sites of LNO treated and control groups were obtained after data filteration. Differential analysis was performed based on t-test. The Gene Ontology and pathway enrichment analysis were analyzed by R package clusterProfiler. In GO terms and Pathways, genes with Q-value less than 0.05 and more than 2 were considered as significantly enriched. GO bar plot was drawn by R package ggplot2 after manual selection of appropriate terms. Heatmap was drawn by R package heatmap and correlation heatmap was drawn by R package corrplot.

Statistical analysis. To assess the statistical significance of differences, ANOVA analysis was performed using GraphPad Prism version 8.2.1 for Windows (GraphPad Software). The statistical tests used to conduct each analysis in the study are described in the corresponding figure legends.

Figures and Tables

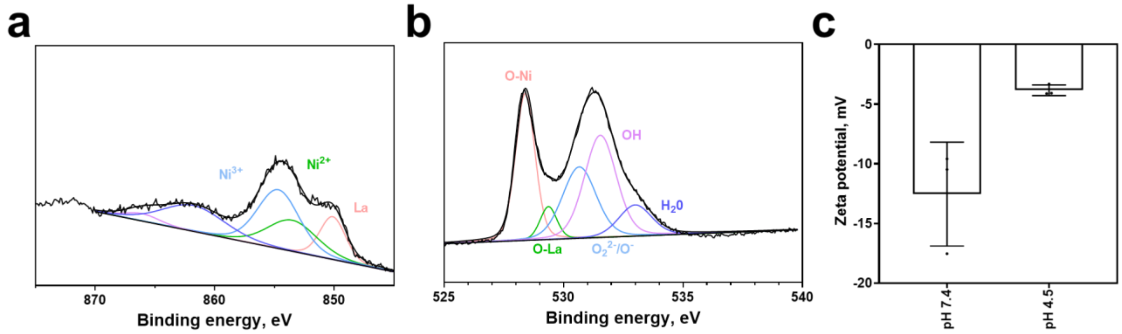

Figure S1. Characterization of LNO. (a, b) XPS spectra of Ni 2p (a) and O 1s (b) of LNO. The binding energies of Ni 2p and O 1s peaks are present at 870-845 eV and 525-535 eV range, respectively. (c) Zeta potential of LNO in PBS in physiological and acidic conditions. Data are expressed as the mean ± standard deviation for triplicated samples (n=3).

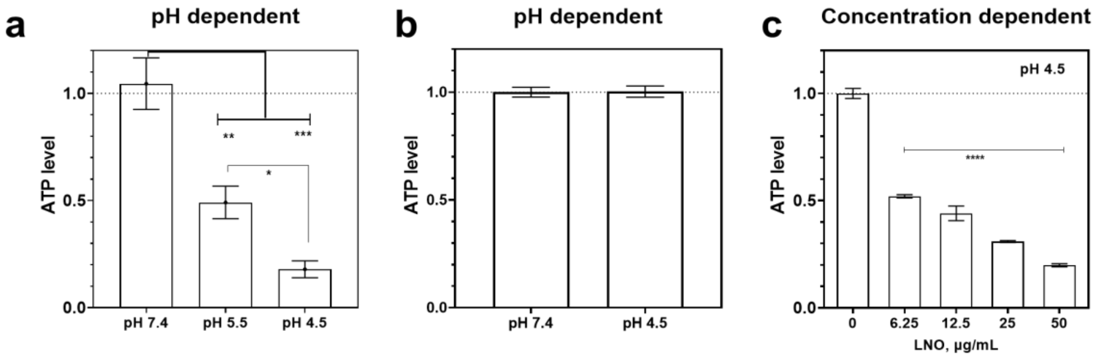

Figure S2. Factors affecting the hydrolysis of ATP. (a, b) Hydrolysis profiles of ATP in physiological and acidic conditions upon incubation with (a) and without (b) LNO. (c) Hydrolysis profiles of ATP in physiological and acidic conditions in a concentration-dependent manner. Statistical significance is calculated by one-way ANOVA with Tukey's multiple comparisons tests; **p* < 0.5; ***p* < 0.01; ****p*<0.001; *****p*<0.0001. Data are expressed as the mean ± standard deviation for triplicated samples (n=3).

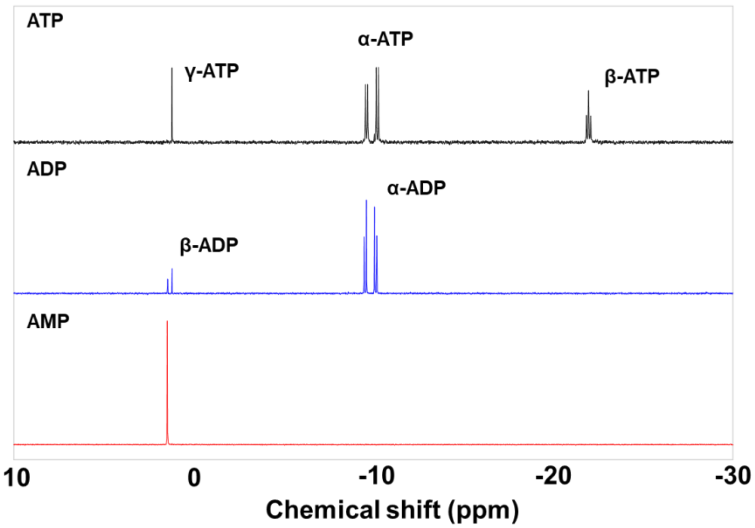

Figure S3. ^{31}P-NMR spectra of solutions of reference samples including ATP, ADP, and AMP.

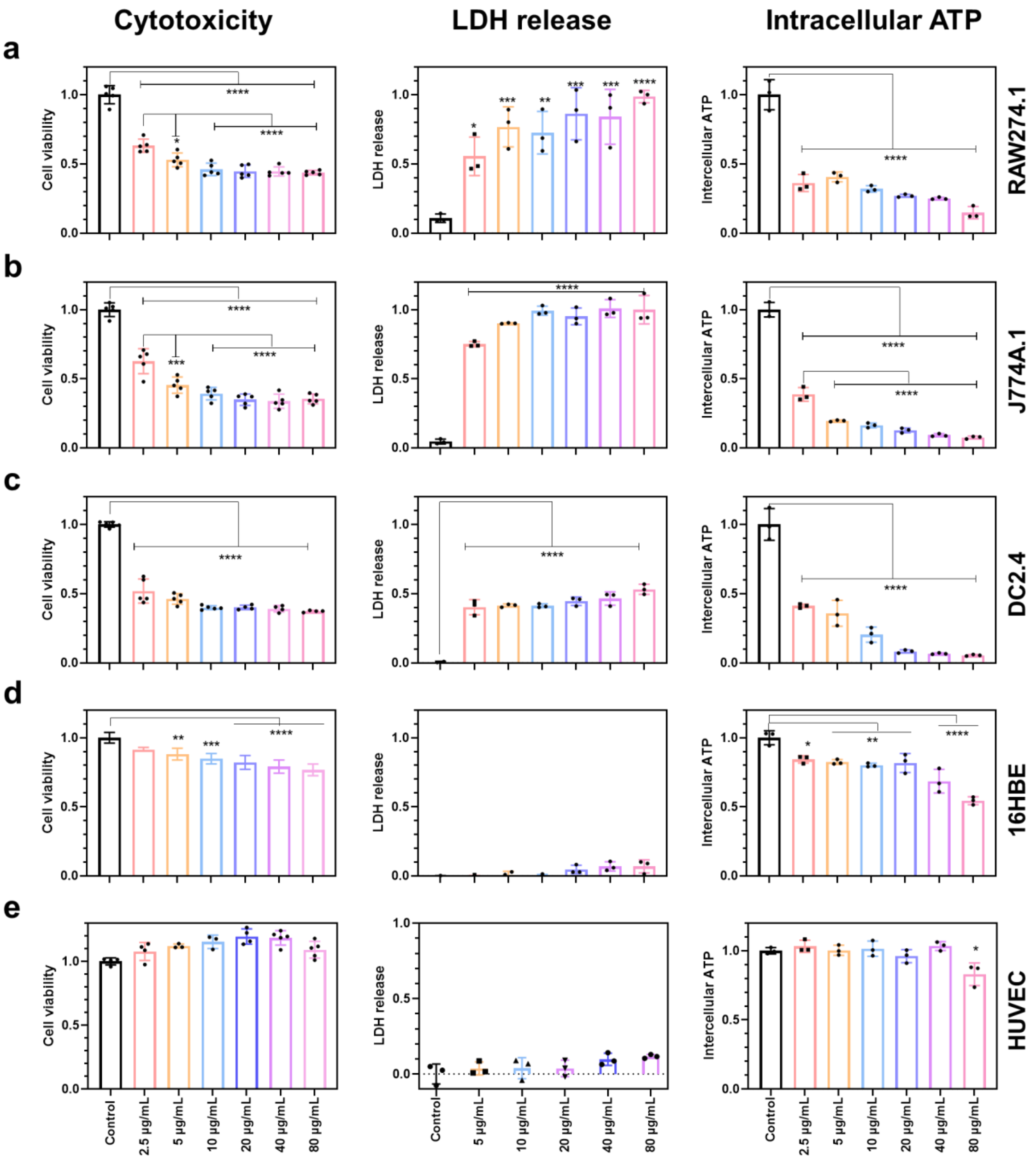

Figure S4. The effect of LNO on the cytotoxicity, LDH release and intracellular ATP from RAW274.1 (a), J774A.1 (b), DC2.4 (c), 16HBE (d) and HUVEC (e) cells after 24 h treatment with LNO at different dosages ranging from 0 to 80 μg/mL. Statistical significance is calculated by one-way ANOVA with Tukey's multiple comparisons tests; *$p < 0.5$, **$p < 0.01$, ***$p<0.001$, ****$p < 0.0001$. Data are expressed as the mean ± standard deviation of replicated samples (n=5) for CCK and triplicated samples for LDH and ATP (n=3) assay kits.

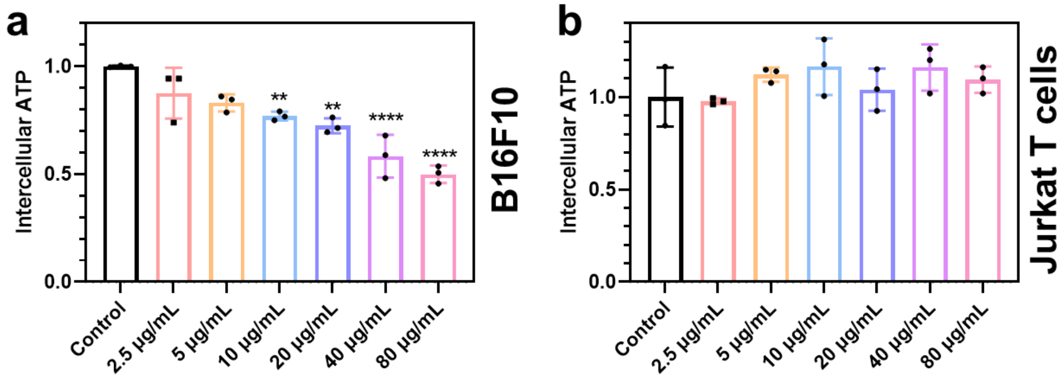

Figure S5. The levels of intracellular ATP from B16F10 (a) and Jurkat T cells (b) after 24 h treatment with LNO at different dosages ranging from 0 to 80 µg/mL. Statistical significance is calculated by one-way ANOVA with Tukey's multiple comparisons tests; *$p < 0.5$; **$p < 0.01$; ***$p<0.001$; ****$p < 0.0001$. Data are expressed as the mean ± standard deviation for triplicated samples (n=3).

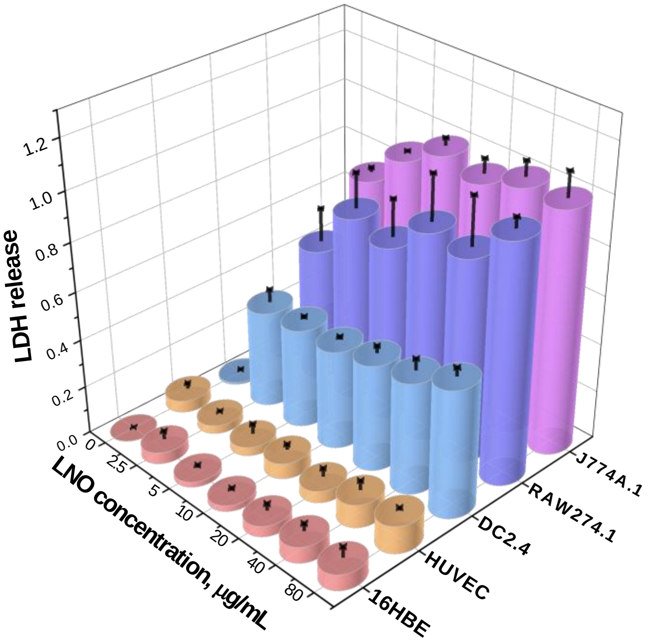

Figure S6. The level of LDH released from cells after 24 h treatment with LNO at different dosages ranging from 0 to 80 μg/mL. Data are expressed as the mean ± standard deviation for triplicated samples (n=3).

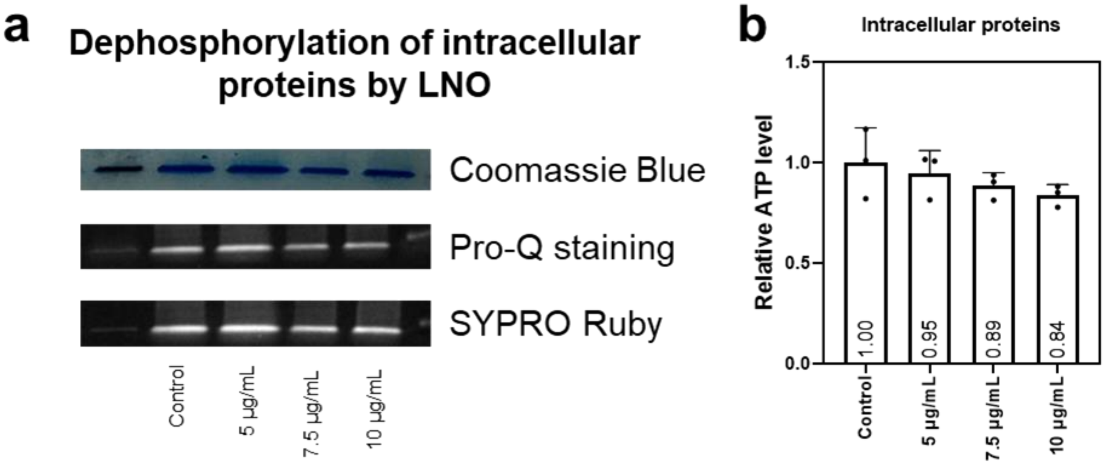

Figure S7. Dephosphorylation of intracellular proteins by LNO. Phosphorylation is determined by Pro-Q-Diamond (phosphor-proteins), and Sypro Ruby and Coomassie Blue staining are used to detect total proteins. RAW264.7 macrophages are treated with LNO at various concentrations for 24 h. Proteins are collected in RIPA buffer supplied with phosphatase and protease inhibitors. Data are expressed as the mean ± standard deviation for triplicated samples (n=3).

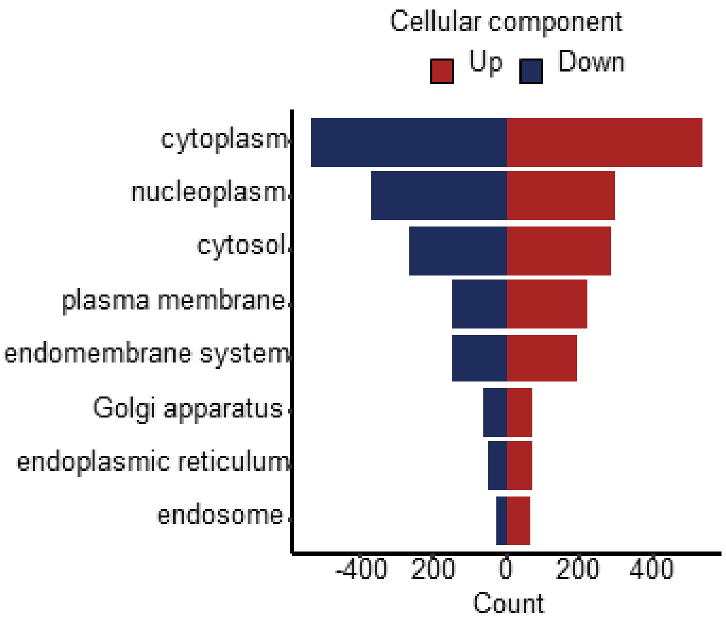

Figure S8. Subcellular localization (cell components enrichment) of significant phosphorylation sites for LNO-treated group compared to control. Upregulated phosphosites are labeled as red, downregulated are labelled as blue.

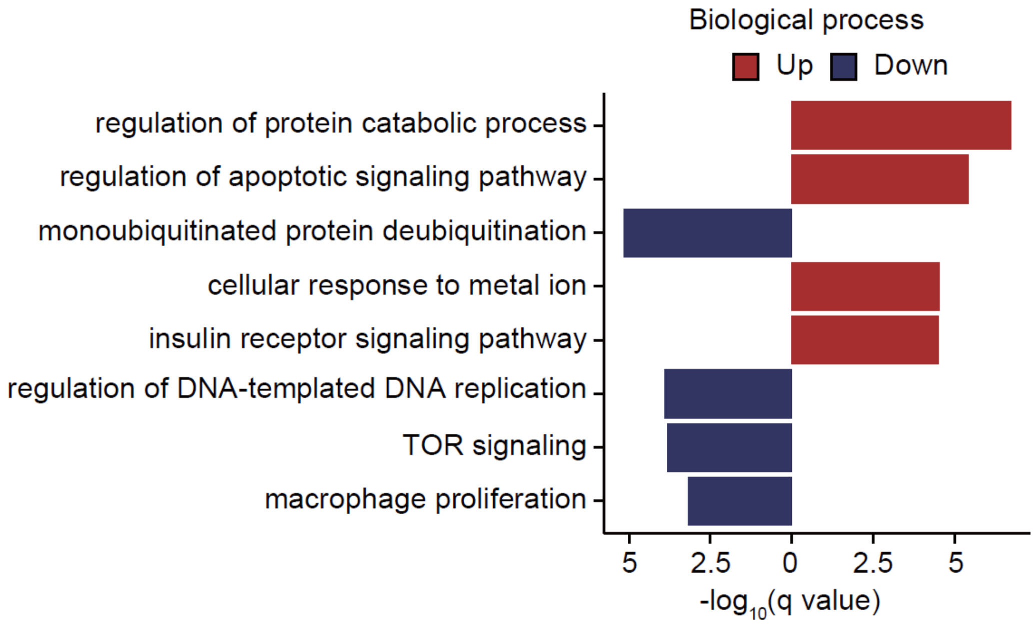

Figure S9. List of up- and down- regulated biological processes for LNO treated group compared to control. Up-regulated phosphosites are labeled as red and down-regulated ones are labelled as blue.

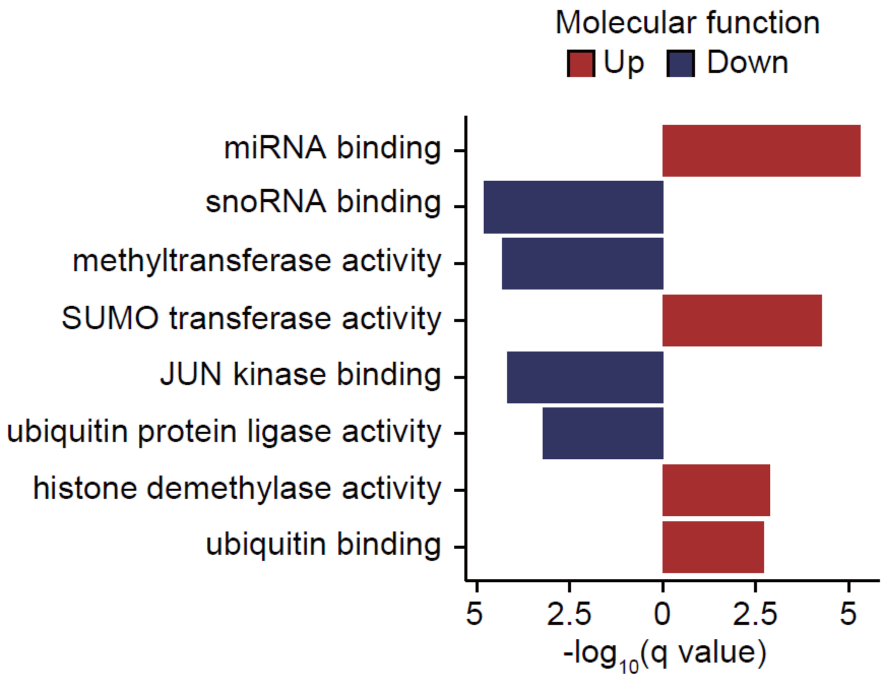

Figure S10. List of up and down regulated molecular functions for LNO-treated group compared to control. Up-regulated phosphosites are labeled as red, and down-regulated ones are labelled as blue.

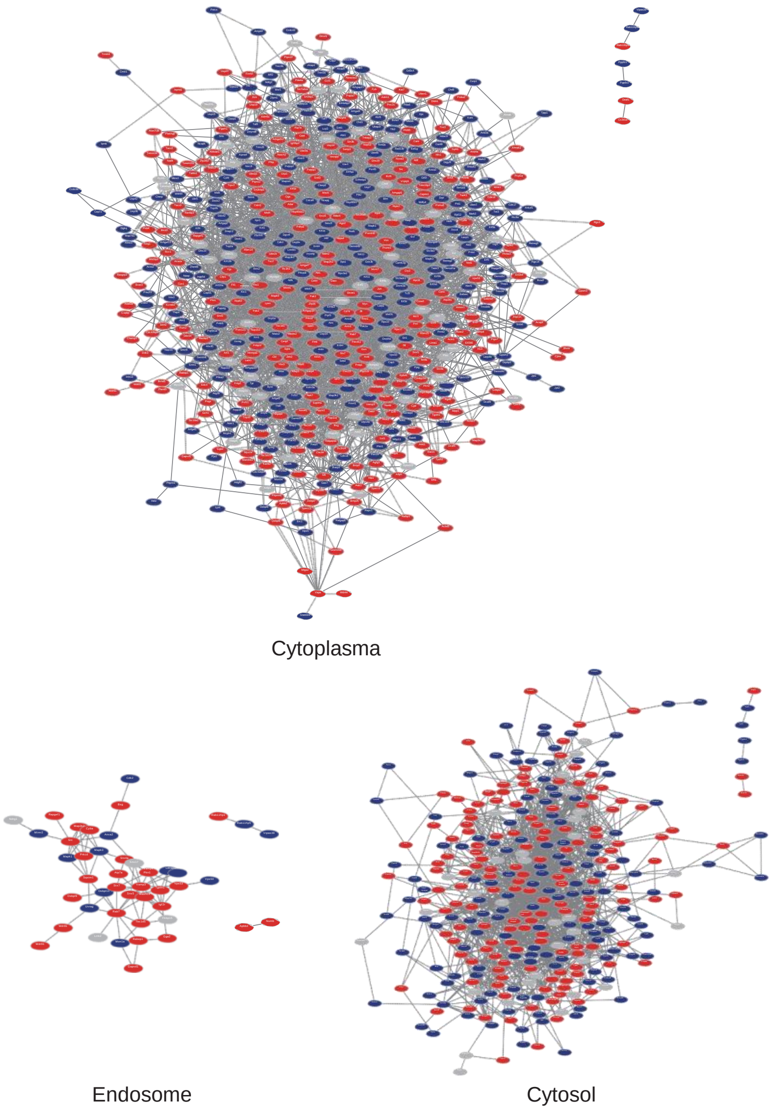

Figure S11. Colocalized subcellular proteins interaction network in screen using STRING upon LNO treatment in cytoplasm, endosome and cytosol. Only text mining, experiments and databases were chosen as interaction sources. The confidence score was set as medium confidence (0.400). The PPI enrichment p-value indicated that these network between differential phosphorylated proteins is significantly enriched than a random set of proteins of the same size and degree distribution drawn from the Mus musculus genome ($p < 1\times10^{-16}$).

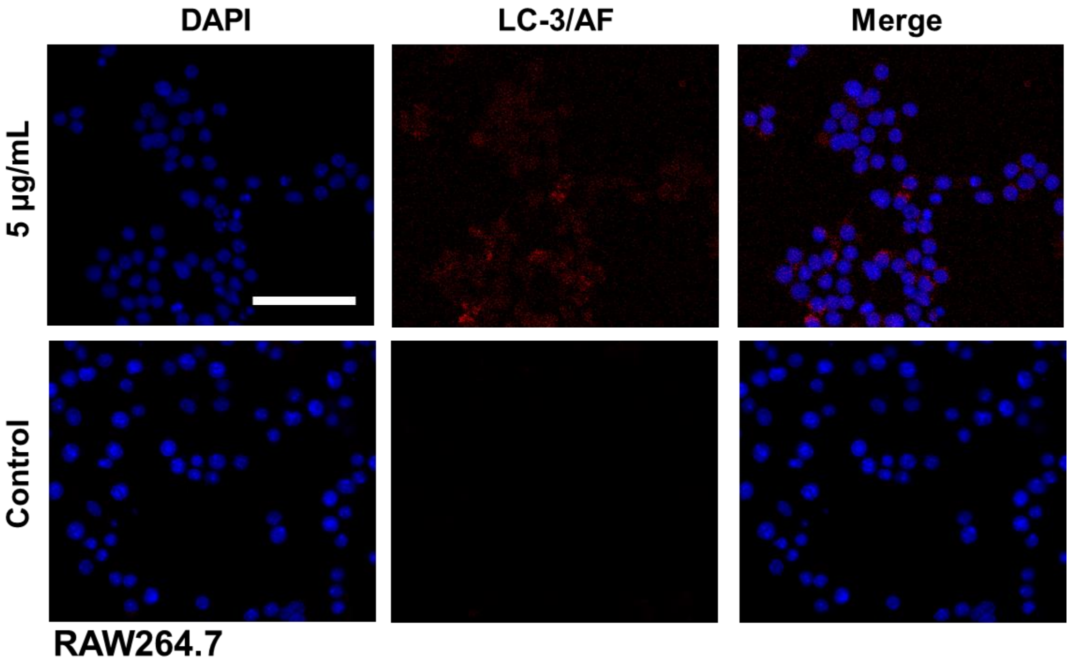

Figure S12. Confocal images of LNO-treated RAW264.7 macrophages. Cell nucleus are stained with DAPI (blue), and macrophages are incubated with LC-3 primary antibody and AF633 secondary antibody (red). The scale bar represents 100 μm.

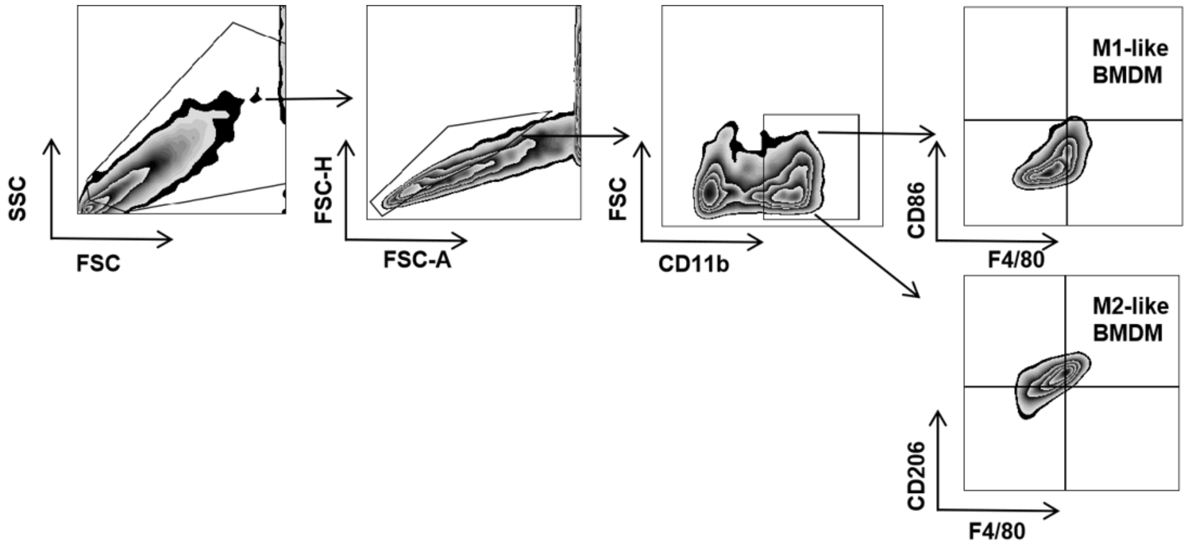

Figure S13. Gating strategy for identifying M1- and M2-like BMDMs.

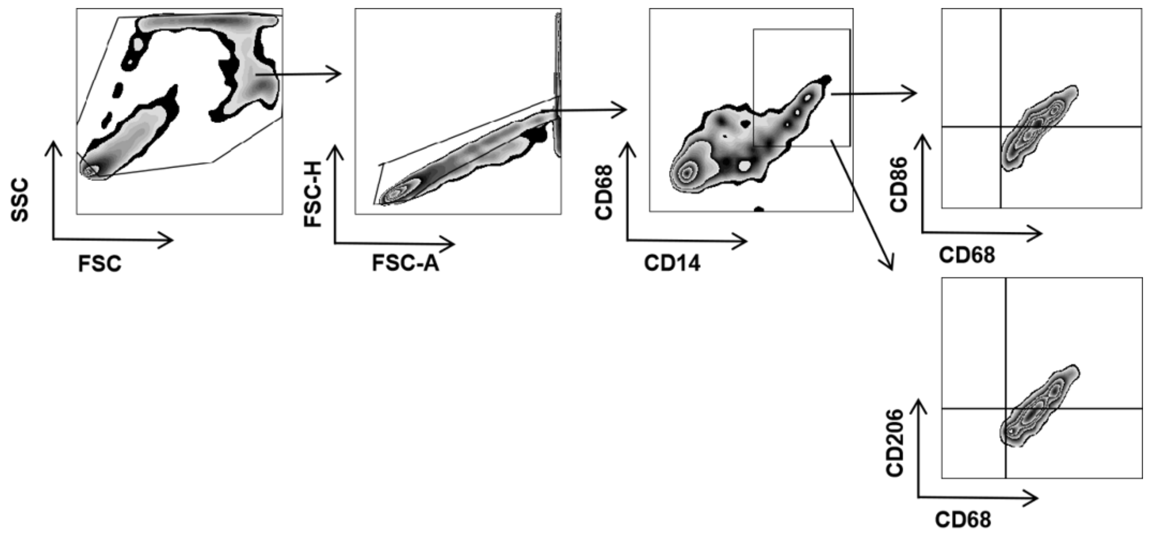

Figure S14. Gating strategy for identifying M1- and M2-like human mononuclear macrophage.

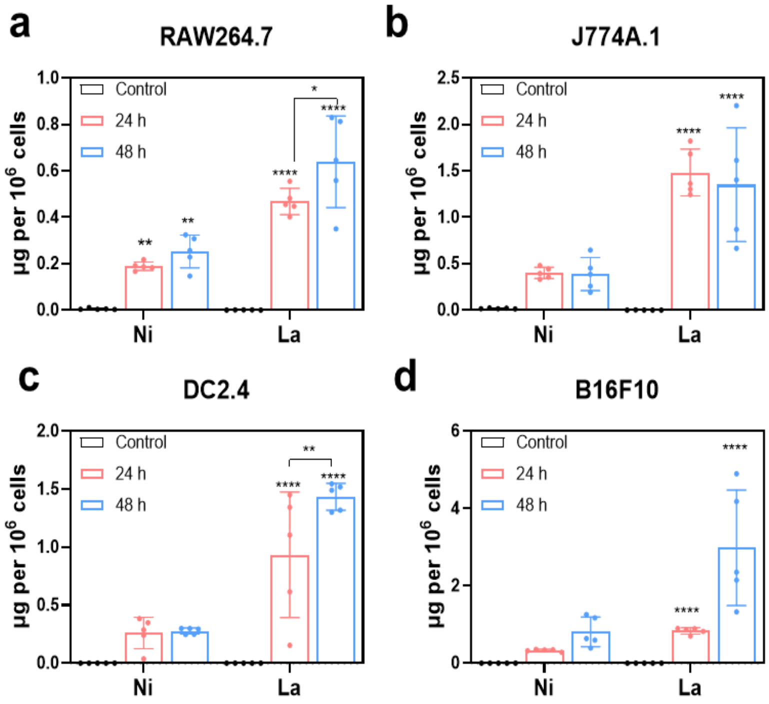

Figure S15. Cellular uptake of LNO in different cells. (a-d) Cellular uptake of LNO by RAW264.7 (a), J774A.1 (b), DC2.4 (c) and B16F10 (d) as determined by ICP-MS. Statistical significance is calculated by one-way ANOVA with Tukey's multiple comparisons tests; n.s., not significant ($p > 0.05$); *$p < 0.05$; **$p < 0.01$; ****$p < 0.0001$. Data are expressed as the mean ± standard deviation for replicated samples (n=5).

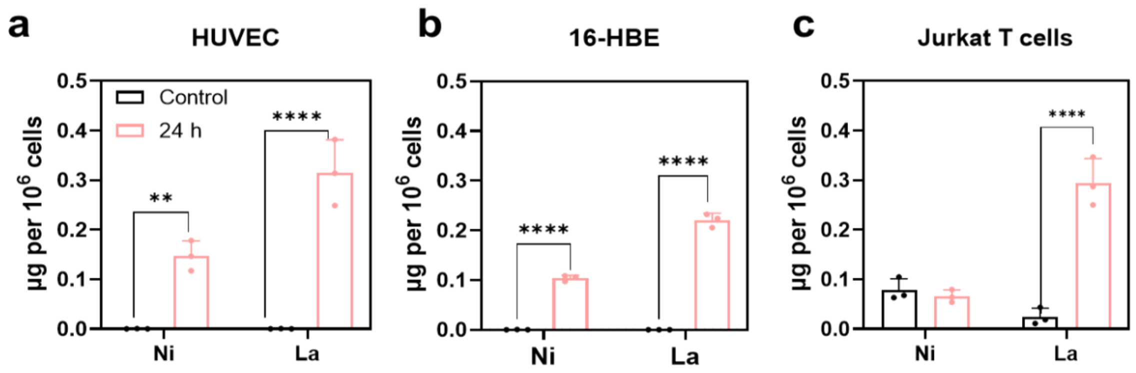

Figure S16. Cellular uptake of LNO in different cells. (a-d) Cellular uptake of LNO by HUVEC (a), 16-HBE (b), Jurkat cells (c) as determined by ICP-MS. Statistical significance is calculated by one-way ANOVA with Tukey's multiple comparisons tests; n.s., not significant (p > 0.05); *p < 0.05; **p < 0.01; ****p < 0.0001. Data are expressed as the mean ± standard deviation for replicated samples (n=3).

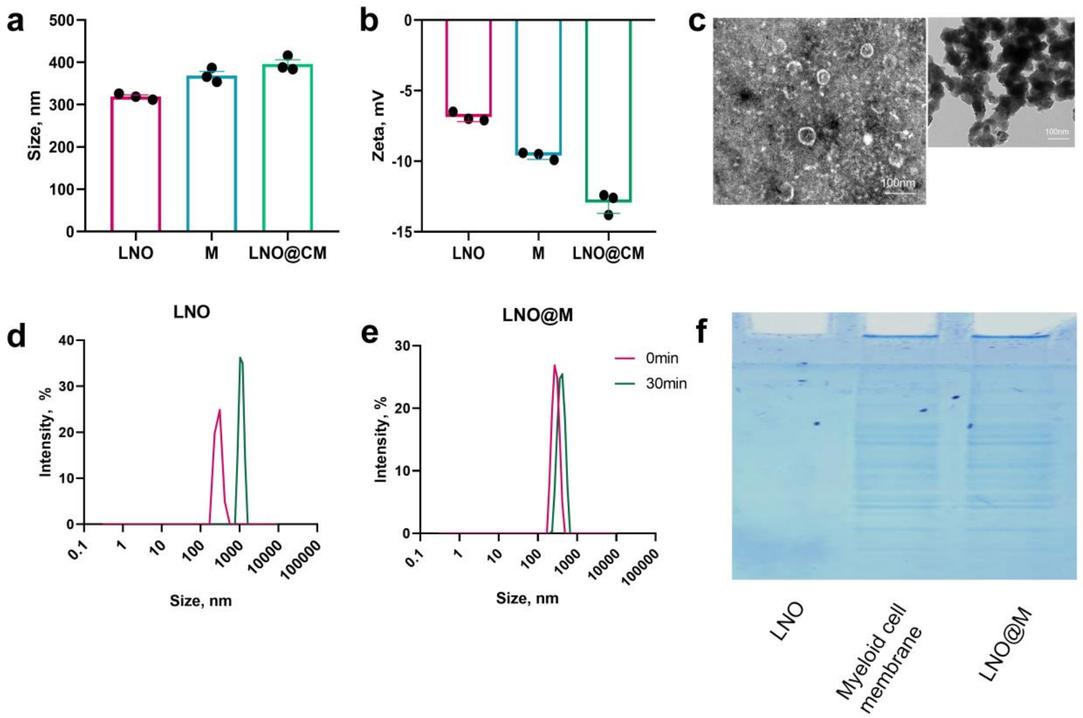

Figure S17. Characterization of myeloid cell membrane-coated LNO@M nanoparticles. (a-f) Size distribution (a), Zeta potential (b), TEM images (c), and size distribution of LNO@M at different time points (d, e) and SDS-PAGE analysis (f). Samples are run at equal protein concentration and gel is stained with Coomassie Blue staining solution of bare LNO and myeloid cell membrane (M) and membrane coated LNO@M nanoparticles. The scale bar represents 100 nm. Data are expressed as the mean ± standard deviation for replicated samples (n=3).

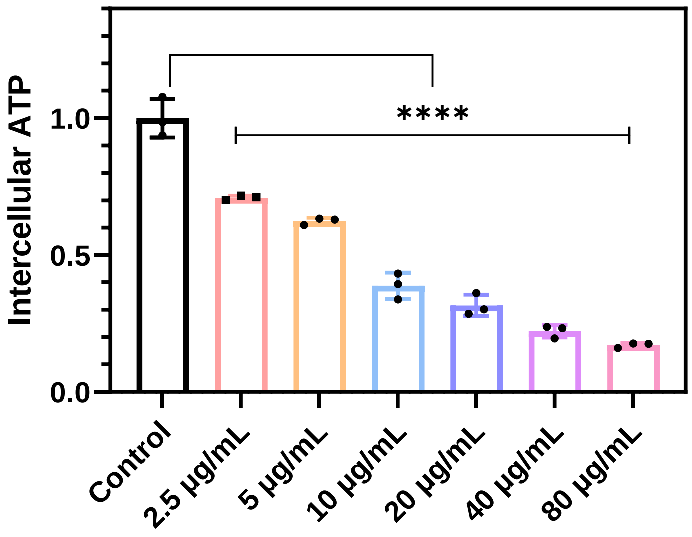

Figure S18. The levels of intracellular ATP from RAW264.7 cells after 24 h treatment with LNO@M at different dosages ranging from 0 to 80 μg/mL. Statistical significance is calculated by one-way ANOVA with Tukey's multiple comparisons tests; ****p < 0.0001. Data are expressed as the mean ± standard deviation of replicated samples (n=3).

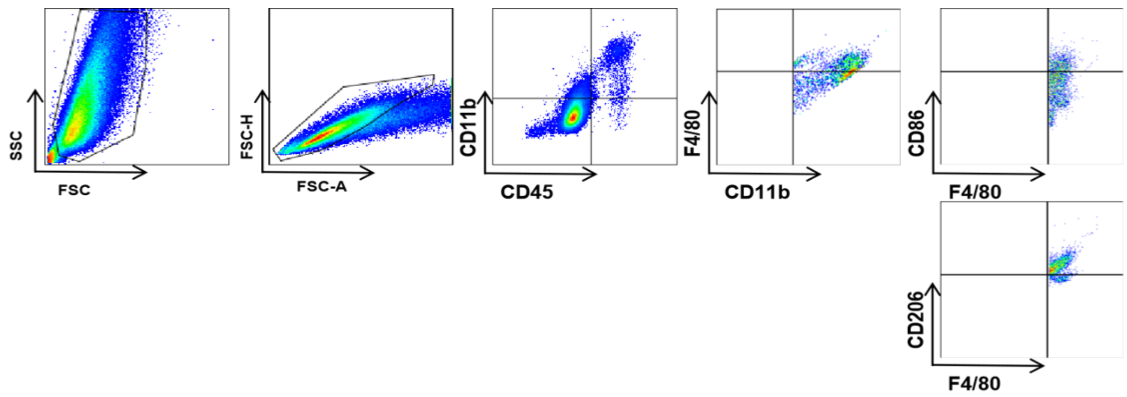

Figure S19. Gating strategy for identifying M1- and M2-like TAMs in vivo.

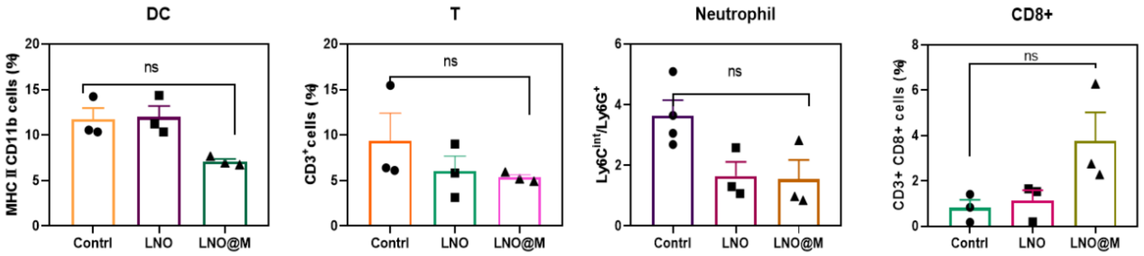

Figure S20. Flow cytometric analysis and quantification of DC and T cells, $CD8^+$ and neutrophil populations in B16 tumors on day 18 post implantation. Statistical significance is calculated by t test; n.s., not significant ($p > 0.05$); *$p < 0.05$. Data are expressed as the mean ± standard deviation of replicated biological samples (n=3).

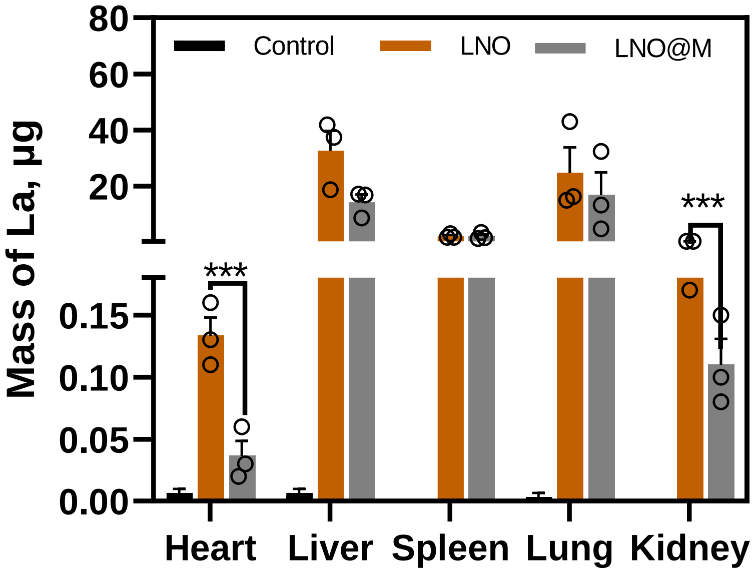

Figure S21. The amount of lanthanum accumulated in the major organs of B16 tumor-bearing C57BL/6 mice. Metal content in the heart, liver, spleen, lung and kidney on day 18 post-treatment by saline, LNO and LNO@M (7.5 mg/kg). Statistical significance is calculated by one-way ANOVA with Tukey's multiple comparisons tests; ***p<0.001. Data are expressed as the mean ± standard deviation of replicated biological samples (n=3).

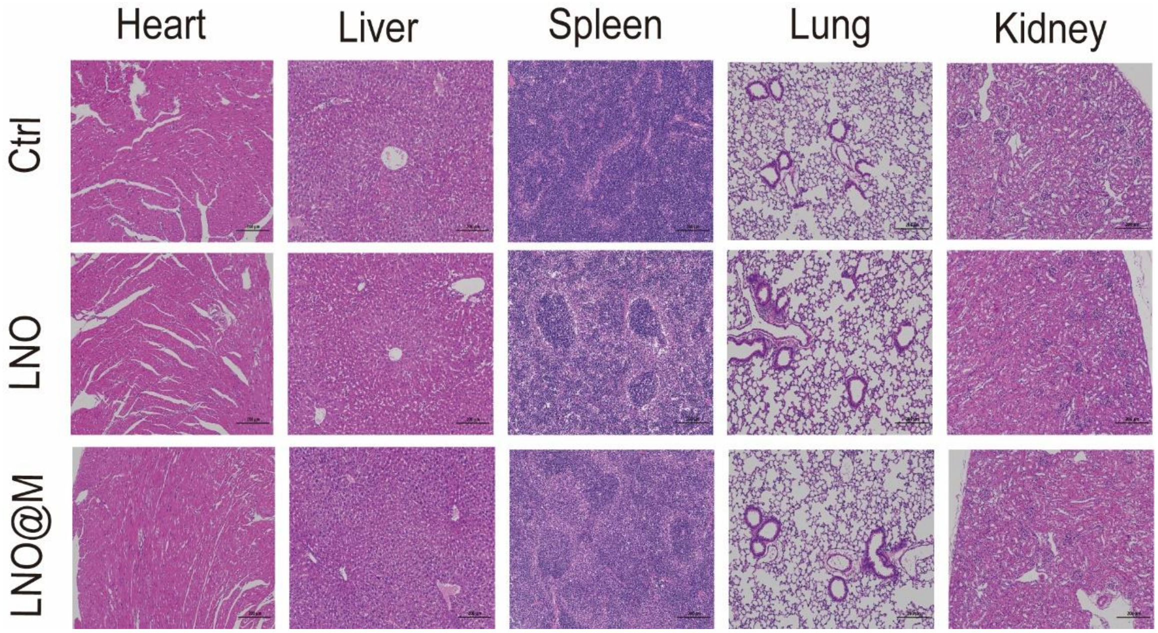

Figure S22. H&E staining images of tissues. Mice are sacrificed upon 18 day-post the intravenous injection with various formulas including blank saline, bare LNO and LNO@M. Scale bar represents 200 μm.

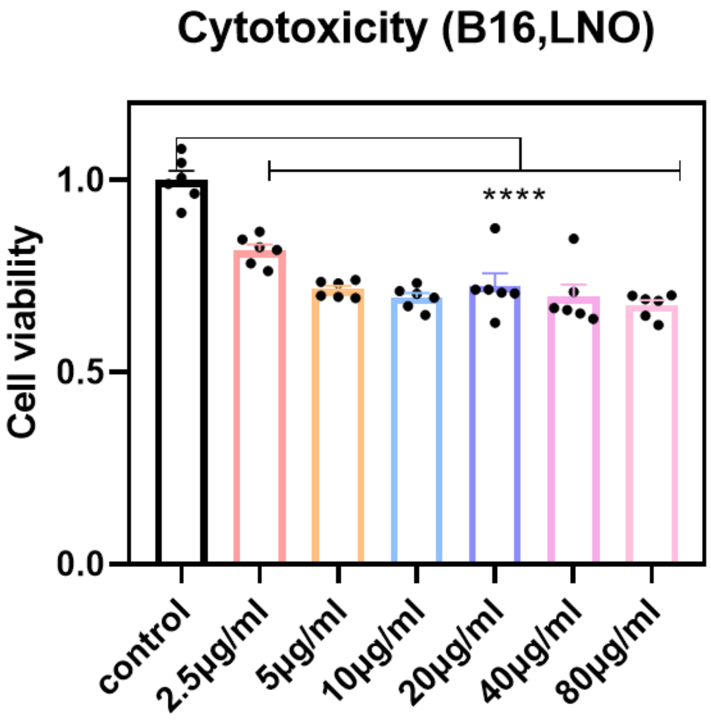

Figure S23. The cell viability of B16F10 cells after 24 h treatment with LNO at different dosages ranging from 0 to 80 μg/mL. Statistical significance is calculated by t tests; ****$p < 0.0001$. Data are expressed as the mean ± standard deviation for replicated samples (n=6).

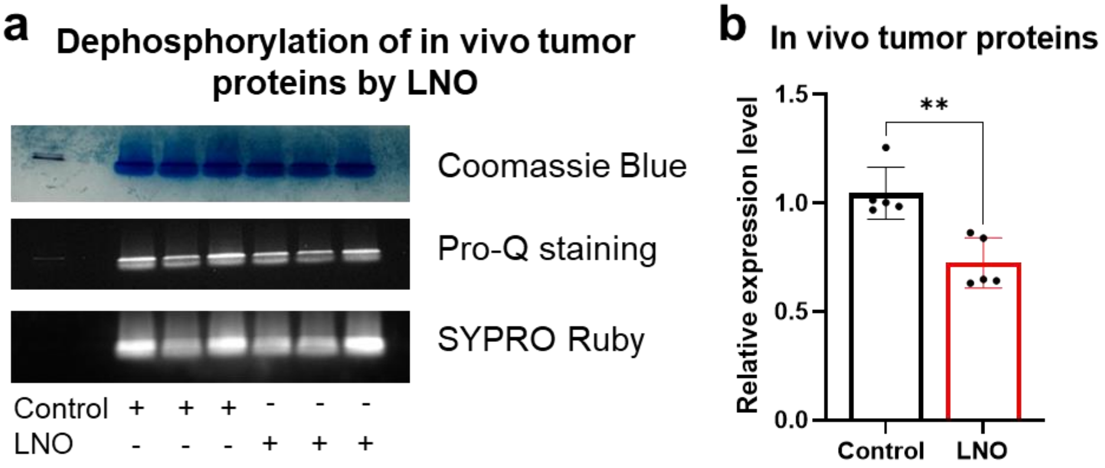

Figure S24. Dephosphorylation of *in vivo* tumor proteins by LNO. (a) Phosphorylation determined by Pro-Q-Diamond (phosphor-proteins), and Sypro Ruby and Coomassie Blue staining for the detection of total proteins. Ex vivo proteins from tumor tissues of B16F10 bearing mice are collected upon treatment with LNO. Proteins of triplicated biological samples are collected in RIPA buffer supplied with phosphatase and protease inhibitors. (b) Expressions of phosphoproteins in control and LNO treated groups are presented. Expressions of triplicated samples from two independent gels are calculated, followed by 31% proteins dephosphorylation upon LNO treatment *in vivo*. Statistical significance is calculated by one-way ANOVA with Tukey's multiple comparisons tests; $^{**}p < 0.01$. Data are expressed as the mean ± standard deviation for replicated samples (n=5).

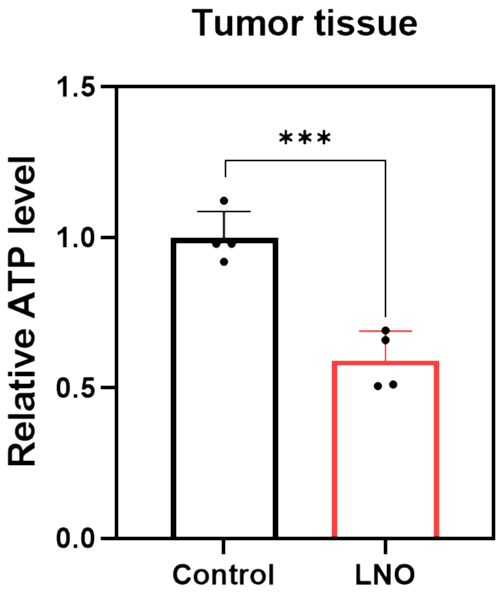

Figure S25. The relative ATP level from tumor tissues of control and LNO treated groups. In detail, 20 mg of tissue sample from untreated control group and LNO treated group were lysed in 200 μL lysis buffer supplied with phosphatase and protease inhibitors. Obtained protein solutions were boiled for 2 minutes to enhance tissue ATP level detection ratio. Statistical significance is calculated by t test; ***p<0.001. Data are expressed as the mean ± standard deviation of replicated biological samples (n=4).

Legends for Movies S1. Nano-CT imaging for intracellular of LNO accumulated in a single RAW 264.7 cell.

Legends for Datasets S1

Table S1. The identified phosphosites of control and LNO-treated RAW 264.7 cells.

Table S2. Up and down regulated phosphosites upon LNO-treatment.

Table S3. Subcellular localization (cell components enrichment) of significant phosphorylation sites for LNO-treated group compared to control in Fig. S8.

Table S4. List of up- and down- regulated biological processes for LNO treated group compared to control in Fig. S9.

Table S5. List of up and down regulated molecular functions for LNO-treated group compared to control in Fig. S10.

Table S6. Analysis of phosphosites on proteins in the autophagy signaling network and several upstream pathways in Fig.3d.

Table S7. Analysis of intracellular dephosphorylation impact on macrophage polarization.

Table S8. Encapsulation efficiency of LNO@M.

References

1. X. Wang, X. J. Gao, L. Qin, C. Wang, L. Song, Y.-N. Zhou, G. Zhu, W. Cao, S. Lin, L. Zhou, K. Wang, H. Zhang, Z. Jin, P. Wang, X. Gao and H. Wei, eg occupancy as an effective descriptor for the catalytic activity of perovskite oxide-based peroxidase mimics, *Nat. Commun.*, 2019, **10**, 704.
2. S. Grimme, S. Ehrlich and L. Goerigk, Effect of the damping function in dispersion corrected density functional theory, *J. Comput. Chem.*, 2011, **32**, 1456-1465.
3. S. Grimme, J. Antony, S. Ehrlich and H. Krieg, A consistent and accurate ab initio parametrization of density functional dispersion correction (DFT-D) for the 94 elements H-Pu, *J. Chem. Phys.*, 2010, **132**, 154104.
4. J. P. Perdew, K. Burke and M. Ernzerhof, Generalized gradient approximation made simple, *Phys. Rev. Lett.*, 1996, **77**, 3865.
5. H. J. Monkhorst and J. D. Pack, Special points for Brillouin-zone integrations, *Phys. Rev. B*, 1976, **13**, 5188.
6. G. Kresse and J. Furthmüller, Efficiency of ab-initio total energy calculations for metals and semiconductors using a plane-wave basis set, *Comput. Mater. Sci.*, 1996, **6**, 15-50.
7. G. Kresse and D. Joubert, From ultrasoft pseudopotentials to the projector augmented-wave method, *Phys. Rev. B*, 1999, **59**, 1758.
8. P. E. Blöchl, Projector augmented-wave method, *Phys. Rev. B*, 1994, **50**, 17953.
9. Y.-D. Zhao, H.-W. An, M. Mamuti, X.-Z. Zeng, R. Zheng, J. Yang, W. Zhou, Y. Liang, G. Qin, D.-Y. Hou, X. Liu, H. Wang, Y. Zhao and X. Fang, Reprogramming Hypoxic Tumor-Associated Macrophages by Nanoglycoclusters for Boosted Cancer Immunotherapy, *Adv. Mater.*, 2023, **35**, 2211332.
10. G. Zhang, Y. Cong, F.-L. Liu, J. Sun, J. Zhang, G. Cao, L. Zhou, W. Yang, Q. Song, F. Wang, K. Liu, J. Qu, J. Wang, M. He, S. Feng, D. Baimanov, W. Xu, R.-H. Luo, X.-Y. Long, S. Liao, Y. Fan, Y.-F. Li, B. Li, X. Shao, G. Wang, L. Fang, H. Wang, X.-F. Yu, Y.-Z. Chang, Y. Zhao, L. Li, P. Yu, Y.-T. Zheng, D. Boraschi, H. Li, C. Chen, L. Wang and Y. Li, A nanomaterial targeting the spike protein captures SARS-CoV-2 variants and promotes viral elimination, *Nat. Nanotechnol.*, 2022, **17**, 993-1003.
11. D. Baimanov, J. Wang, J. Zhang, K. Liu, Y. Cong, X. Shi, X. Zhang, Y. Li, X. Li, R. Qiao, Y. Zhao, Y. Zhou, L. Wang and C. Chen, In situ analysis of nanoparticle soft corona and dynamic evolution, *Nat. Commun.*, 2022, **13**, 5389.
12. Y. Perez-Riverol, J. Bai, C. Bandla, D. García-Seisdedos, S. Hewapathirana, S. Kamatchinathan, Deepti J. Kundu, A. Prakash, A. Frericks-Zipper, M. Eisenacher, M.

Walzer, S. Wang, A. Brazma and Juan A. Vizcaíno, The PRIDE database resources in 2022: a hub for mass spectrometry-based proteomics evidences, Nucleic Acids Res., 2022, 50, D543-D552.
